# Assessing medical devices: a qualitative study from the validate perspective

**DOI:** 10.1017/S0266462324000254

**Published:** 2024-04-24

**Authors:** Bart Bloemen, Wija Oortwijn

**Affiliations:** 1 IQ Health Science Department, Donders Institute for Brain, Cognition and Behaviour, Radboud University Medical Center, Nijmegen, Netherlands; 2 IQ Health Science Department, Research Institute for Medical Innovation, Radboud University Medical Center, Nijmegen, Netherlands

**Keywords:** medical devices, health technology assessment, values, commitments, normativity

## Abstract

**Objectives:**

Our objective was to explore procedures and methods used at health technology assessment (HTA) agencies for assessing medical devices and the underlying views of HTA practitioners about appropriate methodology to identify challenges in adopting new methodologies for assessing devices. We focused on the role of normative commitments of HTA practitioners in the adoption of new methods.

**Methods:**

An online survey, including questions on procedures, scoping, and assessments of medical devices, was sent to members of the International Network of Agencies for Health Technology Assessment. Interviews were conducted with survey respondents and HTA practitioners involved in assessments of transcatheter aortic valve implantation to gain an in-depth understanding of choices made and views about assessing medical devices. Survey and interview questions were inspired by the “values in doing assessments of health technologies” approach towards HTA, which states that HTA addresses value-laden questions and information.

**Results:**

The current practice of assessing medical devices at HTA agencies is predominantly based on procedures, methods, and epistemological principles developed for assessments of drugs. Both practical factors (available time, demands of decision-makers, existing legal frameworks, and HTA guidelines), as well as commitments of HTA practitioners to principles of evidence-based medicine, make the adoption of a new methodology difficult.

**Conclusions:**

There is a broad recognition that assessments of medical devices may need changes in HTA methodology. In order to realize this, the HTA community may require both a discussion on the role, responsibility, and goals of HTA, and resulting changes in institutional context to adopt new methodologies.

## Introduction

Health technology assessment (HTA) aims to *inform* decision-makers by assessing the *potential value* of health technologies (1). Therefore, HTA practitioners (those responsible for conducting assessments, including scoping, collecting, synthesizing, and interpreting available evidence) need to identify evidence that can answer policy-relevant questions about the potential value of health technology, requiring decisions on which information can be regarded as reliable and relevant. Current discussions about appropriate HTA methodology for assessing (high-risk) medical devices show that this is not an easy task. Based on differences between medical devices and drugs, scholars argue that HTA methodology for medical devices should be adapted to 1) *integrate other types of evidence* (e.g., real-world evidence) to address the lack of evidence from randomized clinical trials and capture the impact of iterative developments of devices on outcomes; 2) *broaden the scope of assessments* to capture organizational aspects (e.g., impact on healthcare capacity); and 3) *involve stakeholders in assessments* (e.g., making methodological decisions) to address context-dependence of outcomes and gather information on user experiences and preferences (2–8).

Despite these calls to assess medical devices differently, previous studies have shown that HTA agencies use similar methodologies for assessing drugs and medical devices (2;4;5;9;10). Although practical reasons like capacity problems and existing regulatory frameworks contribute to this uniformity, we argue that *normative commitments* of HTA agencies and practitioners also play a role. Inspired by the “values in doing assessments of health technologies” (VALIDATE) approach, which emphasizes that the relevance and meaning of evidence considered in HTA depends on underlying values, we reasoned that both the value perspectives of stakeholders and HTA practitioners are instrumental in conducting assessments (11;12). This implies that the activities of HTA agencies and practitioners are not solely guided by established HTA guidelines but are also influenced by practitioners’ views on how HTA can improve outcomes of health technology for society. Given that HTA is often presumed to provide information about the *public value* of health technology, transcending particular interests, HTA practitioners and agencies are committed to methodological principles presumed to guarantee a *neutral* or *unbiased* evidence base for decision-makers (13–15). These commitments may conflict with new types of evidence, outcome measures, and methodologies proposed for assessing medical devices.

To explore the significance of these commitments, besides practical challenges, in the adoption of new methodology (e.g., real-world data, stakeholder involvement) for (high-risk) medical device assessments, we conducted a survey and interview study among relevant HTA agencies. Our objective was to map the procedures and methodologies currently used by these HTA agencies and to retrieve the views of HTA practitioners about the role of HTA, stakeholder involvement, and appropriate evidence in HTA.

## Methods

We used a semi-structured survey to gather information on the current practice of assessing (high-risk) medical devices by HTA agencies (i.e., legal frameworks, procedures, and methods). We defined high-risk medical devices as Class IIb and Class III medical devices according to the European Regulation on Medical Devices – Regulation (EU) 2017/745. Additionally, via semi-structured interviews with HTA practitioners, we explored, building on previous findings in the literature, whether changes in HTA methodology may conflict with their views (13). Specifically, we were interested in their perspectives on the role of HTA in decision-making, their responsibilities in the conduct of HTA, stakeholder involvement, and what constitutes appropriate evidence, particularly for assessing medical devices. Both survey and interview questions, inspired by the VALIDATE approach and literature on HTA for medical devices, also delved into the value-laden aspects of HTA procedures and methodology. See also Supplementary Figure 1 for a schematic illustration of the qualitative approach taken in this study.

### Survey

The online survey was developed based on our previous work regarding deliberative HTA processes (targeting stakeholder involvement), normative analysis, and desk research on challenges in assessing medical devices (2–10;12;16;17). Questions focused on *institutional context* and *current HTA processes*, *scoping*, and *assessing medical devices* (the types of evidence used, aspects assessed, stakeholder involvement). A draft version was tested by an HTA practitioner at a national HTA agency from our network. Based on the received feedback, minor changes were introduced to clarify questions. The survey (and invitation email) is provided as Supplementary file 1.

We invited members of the International Network of Agencies for Health Technology Assessment (INAHTA), except research organizations and regulatory agencies (*n* = 3), and one institute which we know does not assess medical devices. We targeted specific persons known from our networks and/or who assess medical devices; otherwise, contact persons mentioned on the INAHTA website (www.inahta.org) were approached. Data collection occurred via the online tool CheckMarket, between January and February 2023, including two biweekly reminders. We asked respondents for consent to analyze results and assured confidentiality (no attribution is made to specific persons). We also asked for consent to contact them for an interview.

Descriptive statistics (frequencies presented as percentages) derived from the CheckMarket tool were used to summarize findings. When needed, websites, literature, and publicly available guidelines and HTA reports from HTA agencies (retrieved by manually searching on their websites) were reviewed to clarify responses and gain an in-depth understanding of processes and methodology used for assessing medical devices, see also Supplementary file 2.

### Interviews

We invited (via email) HTA practitioners who responded to the survey and indicated to be contacted, and specifically invited HTA practitioners involved in assessing Transcatheter Aortic Valve Implantation (TAVI) to explore choices made in real-world assessments. TAVI was chosen as an example because it is a high-risk medical device that has already been implemented in clinical practice, and full HTAs are conducted in different jurisdictions. It is a minimally invasive technology aimed at inoperable patients with symptomatic severe aortic valve stenosis. Since its Conformité Européene (CE) marking in 2007, usage has expanded to patients at high, intermediate, and low surgical risk. We focused on assessments of TAVI for patients at low risk for surgical complications (i.e., eligible for the standard treatment, surgical aortic valve replacement [SAVR]), which became standard care for patients 75 years old and above (18). In November 2022, the HTA database (https://database.inahta.org/) was used to search for full HTA reports using the MeSH term “Transcatheter Aortic Valve Replacement,” which retrieved available HTA reports (on TAVI for low-risk patients) from Health Information and Quality Authority – HIQA (Ireland), Ontario Health (Canada), and the Norwegian Institute of Public Health (19–21). In addition, a manual search retrieved a report by Haute Autorité de Santé (France) (22).

We developed a semi-structured interview guide based on relevant literature on normativity in HTA, challenges in assessing medical devices/TAVI, and the VALIDATE approach. Interviews comprised three parts: (i) professional background, experience, and current position of the HTA practitioner; (ii) questions on context and decisions made in developing the respective HTA report on TAVI, or questions to clarify answers given to survey questions; (iii) personal views of the HTA practitioner on roles and responsibilities of HTA, and methodological issues in assessments of medical devices. The interview guide was iteratively updated based on experiences with conducting the interviews. Given the explorative nature of our study, data saturation was not a target.

The lead author (B.B., PhD candidate in HTA) conducted online interviews (using Microsoft Teams) between February and May 2023, with a duration of 1 to 1.5 hours. All interviews were audio-recorded and summarized; interviewees were asked to provide feedback on the summary to clarify any misunderstandings. Prior to participation, oral consent was obtained from all interviewees, who were informed about the study objectives through invitation mail and the concept interview guide.

More information about the preparation of interviews, and the interview guide, can be found in Supplementary file 3.

The basis for analyzing the interviews was the updated summaries (based on feedback from the interviewees), including information retrieved from the websites of respective HTA agencies, HTA reports, and publicly available guidelines. Thematic analysis was used, which is a method for identifying, analyzing, and reporting themes within the data. Because interviews were conducted to provide in-depth information, complementary to the surveys, about the context and reasons (including views of HTA practitioners) behind current processes and methodology for assessing medical devices (see also Supplementary Figure 1), main themes from the survey (scoping, types of evidence, aspects of devices being assessed, stakeholder involvement) were the starting point for analyzing the interviews. The lead author used a process of inductive comparison and reasoning to identify subthemes that reflect the content of conducted interviews.

The consolidated criteria for reporting qualitative research (COREQ) checklist was used to ensure that the methods, results, and discussion were reported appropriately (23).

## Results

### Study participants

We invited fifty contact persons of INAHTA member agencies, of which twenty-two (response rate of 44 percent) responded to the survey. Two respondents answered less than 50 percent of the main questions and were excluded from the analysis. In addition, five respondents were excluded as they were not involved in the assessment of medical devices. In total, we analyzed fifteen survey responses, including twelve fully completed surveys and three agencies that provided meaningful answers (answering more than 50 percent of questions on either scoping and/or assessment). Among these, eight were willing to be interviewed (53 percent).

Four accepted our invitation for an interview (50 percent) from HTA agencies in the Netherlands, Spain, Taiwan, and Colombia. Of the authors of the four retrieved HTA reports on TAVI who were invited for an interview (*n* = 9), two accepted our invitation, one did initially agree to be interviewed but did not respond after sending multiple reminders to set an interview date, one declined participation, two referred to a co-author, and three did not respond at all. When an author of an HTA report on TAVI accepted the invitation, other authors of the same HTA report were not invited.

Table 1 provides an overview of participating HTA agencies. Additional information about interview participants is reported in Supplementary Table 1. Most participating agencies are governmental institutions (29 percent) or institutes with a government function (47 percent, independent from a Ministry of Health), advising policymakers on national policy decisions (e.g., allocation of public resources, reimbursement by health insurance) on medical devices.

**Table 1.** Overview of HTA agencies that (partially) completed the survey and/or participated in the interviews

Institution, country/region	Type of institution^a^	Completed the survey?	Participated in interviews?
Avalia–t/ACIS, Spain (Galician region)	3	Yes	Yes (on medical devices)
AQuAS, Spain, Catalonia	3	Yes	No
CADTH, Canada	4	Yes (partial response)	No
CDE/HTA, Taiwan	2a	Yes	Yes (on medical devices)
FOPH, Switzerland	2a	Yes	No
G–BA, Germany	5	Yes	No
Health Technology Wales, Wales	4	Yes (partial response)	No
IECS, Argentina	1	Yes	No
IETS, Colombia	4	Yes	Yes (on medical devices)
IQWiG, Germany	4	Yes	No
MaHTAS, Malaysia	2a	Yes	No
NECA, South Korea	4	Yes	No
NIPH, Norway	2a	Yes	No
SR–NRCHD, Kazakhstan	2a	Yes (partial response)	No
ZIN, The Netherlands	4	Yes	Yes (on medical devices)
Ontario Health, Canada	4	No	Yes (on TAVI)
HIQA, Ireland	4	No	Yes (on TAVI)

a Categorization based on Fuchs et al. 2017: 1 = independent academic research entity, 2 = Governmental institutions (a. national, b. regional), 3 = Regional Ministries of Health/Social Affairs including a related department, 4 = Independent entities with function as a governmental institution, 5 = Non-departmental public body with legislative function.

Abbreviations: Avalia-t/ACIS, Unidad de Asesoramiento Científico-técnico (Avalia-t), Axencia Galega de Coñecemento en Saúde (ACIS); AQuAS, Agència de Qualitat I Avaluació Sanitàries de Catalunya; CADTH, Canadian Agency for Drugs and Technologies in Health; CDE/HTA, Center for Drug Evaluation Health Technology Assessment; FOPH, Federal Office of Public Health; G-BA, Gemeinsamer Bundesausschuss; IECS, Instituto de Efectividad Clínica *y* Sanitaria; IETS, Instituto de Evaluación Tecnológica en Salud; IQWiG, Institut für Qualität und Wirtschaftlichkeit im Gesundheitswesen; MaHTAS, Malaysian Health Technology Assessment Section; NECA, National Evidence-based healthcare Collaborating Agency; NIPH, Norwegian Institute of Public Health; SK-NRCHD, Salidat Kairbekova National Research Center for Health Development; ZIN, Zorginstituut Nederland; HIQA, Health Information and Quality Authority.

### Institutional context, procedures for assessing medical devices

Survey respondents and interviewees were asked about how assessments of medical devices are initialized and differences with HTA processes for drugs (see Supplementary Tables 1 and 2).

In general, agencies have similar procedures for assessing devices and drugs, but processes may differ in duration, initialization of assessments, and evidential requirements, being more heterogeneous for devices. The definition of medical devices varies widely: five agencies use EU directives that include specific definitions of (classes of) medical devices, three agencies use a definition from their national law, while five agencies report a broader definition of *health technology* that includes devices.

When a medical device is introduced to a market (after regulatory approval), HTA agencies are mostly asked to conduct assessments that inform reimbursement decisions at the request by decision-makers (73 percent), followed by an application of the manufacturer and identification via horizon scanning (47 percent). Although there are experiments involving stakeholders in deciding which devices need an assessment, this is often limited to proposing topics or providing feedback on a draft HTA protocol, and the final decision rests with decision-makers and sometimes HTA practitioners. Interviewees also mentioned that decision-makers’ needs often determine which assessments are initiated (see also Table 3).

### Scoping

Nine survey respondents (60 percent) reported that their agency has (publicly available) guidelines or documents on scoping applicable to medical devices, see Table 2. Guiding principles of the scoping process are transparency (78 percent), overarching goals of the HTA agency or healthcare system, impartiality, consistency, verifiability (all 67 percent), whereas inclusivity (44 percent), timeliness (44 percent) and efficiency (33 percent) are less frequently mentioned. Scoping often focuses on defining the health technology and its comparators needing an assessment (67 percent), whereas defining the health problem is rarely the objective of scoping (22 percent).

**Table 2.** Overview of answers provided to survey questions on scoping

Question	Answers		Percentage
*Are guidelines/documents describing the process of scoping applicable to the evaluation of high–risk medical devices present in your country/region?* (*n* = 15)	Present and publicly available		27%
	Present but not publicly available		33%
	Not present		40%
*What are the guiding principles of the scoping process described in the guidelines?* [multiple answers possible] (*n* = 9)	Transparency		78%
	Overarching goals of HTA agency or health system		67%
	Impartiality		67%
	Consistency		67%
	Verifiability		67%
	Inclusivity		44%
	Timeliness		44%
	Efficiency		33%
*What is the main focus of the scoping process described in the guidelines?* (*n* = 9)	Defining the health technology and the alternative technology(s) against which the health technology under assessment should be compared		67%
	Defining to what extent the health problem under study can be addressed (i.e., are non–technological interventions that could be proposed to address the health problem being considered)		22%
	Other, please specify: – In relation to the health condition, we used to define the baseline characteristics of the population; moreover, we defined the outcomes that will be assessed in the report (*n* = 1)		11%
*How are stakeholders selected to be involved in the scoping process (if described in the guidelines)?* (*n* = 8)	By invitation or appointment (closed procedure)		50%
	Using a hybrid approach		38%
	Open to all who qualify (application process)		13%
	Open to all (public call)		0%
	Nominated by relevant interest groups (nomination process)		0%
*Which input is requested from stakeholders in the scoping process?* [multiple answers possible] (*n* = 8)	Background information provided by stakeholders (e.g., experiential knowledge that can help in defining the research question, ideas about the plausibility of different interventions in addressing the health problem, different views on how to define the health problem)		88%
	The contribution of stakeholders is primarily focused on providing value perspectives and selecting relevant outcomes		63%
	Stakeholders are explicitly involved in determining the objectives of the assessment		50%
*Which stakeholders are explicitly involved via consultation (i.e., structured process to collect feedback among groups of stakeholders on specific decisions via, e.g., surveys, interviews, expert panels, patient testimonies); and which stakeholders are involved via participation (i.e., active engagement in deliberations and open exchange on argumentation and evidence)?* [multiple answers possible] (*n* = 8)	**Stakeholder**	**Consultation (relative position)**	**Participation (relative position)**
	Providers of care (e.g., clinician, nurse, hospital board member, and so)	88% (1)	88% (1)
	Experts in medicine	88% (1)	88% (1)
	Patient’s organization	75% (2)	75% (2)
	Experts in (health) economics	63% (3)	88% (1)
	Policymakers	63% (3)	50% (4)
	Experts in epidemiology	50% (4)	63% (3)
	Manufacturers	50% (4)	50% (4)
	Experts in ethics	38% (5)	50% (4)
	Experts in healthcare administration	38% (5)	38% (5)
	Payers/purchasers (e.g., health insurers, HMOs, and so)	38% (5)	0% (8)
	Patients with the disease but not yet treated	25% (6)	13% (7)
	Patients with the disease and already treated with the comparator	25% (6)	25% (6)
	Patients treated with the new intervention	25% (6)	13% (7)
	Informal caregivers	25% (6)	13% (7)
	Experts in patient/public involvement	25% (6)	25% (6)
	Experts in bioengineering	25% (6)	38% (5)
	Experts in statistics	25% (6)	25% (6)
	Experts in law	13% (7)	38% (5)
	Experts in psychology	13% (7)	13% (7)
	Public/(organized) group of citizens	13% (7)	13% (7)
*Which tool(s) are used for scoping (if described in guidelines)? [multiple answers possible]* (*n* = 8)	Population intervention comparators outcomes (PICO) tool		100%
	Technology indication comparison outcome (TICO) tool		13%
	Other, please specify: – We also use the PICOD (D = design) tool (*n* = 1)		13%
*Which methods are used for selecting comparators and outcome measures to be considered in an assessment?* [multiple answers possible] (*n* = 8)		**Comparators (relative position)**	**Outcome measures (relative position)**
	Literature or document review	100% (1)	88% (1)
	Interviews with health professionals relevant to the disease under study	63% (2)	50% (2)
	Interviews with other relevant experts	50% (3)	25% (4)
	Focus groups with a mix of relevant experts, including health professionals and/or patients	38% (4)	38% (3)
	Interviews with patients suffering from the disease under study	25% (5)	25% (4)
	Surveys of relevant stakeholders	25% (5)	38% (3)
	Other, please specify: – Interviews used to be done by telephone or email (*n* = 1) – We have an evidence assessment group and a patient and public involvement group that consider and agree on relevant outcomes and methods (*n* = 1)	25% (5)	25%
	Focus groups with health professionals relevant to the disease under study	13% (6)	25% (4)
	Focus groups with patients suffering from the disease under study	13% (6)	13% (5)
	Focus groups with other relevant experts	13% (6)	25% (4)

Eight agencies (53 percent) have a description of stakeholder involvement included in their guidelines for scoping. Input requested from stakeholders primarily provides background information (88 percent) and information on their value perspectives and ideas about relevant outcome measures (63 percent). Stakeholders are recruited by invitation (50 percent) or a combination of closed and open procedures (38 percent). The stakeholders mostly involved in scoping are providers of care, experts in medicine, patients’ organizations, experts in health economics, and policymakers, whereas the involvement of patients themselves (not represented via a patients’ organization), informal caregivers, and the public (organized group of citizens) is low (25 percent or less). Some groups of stakeholders are mostly involved in a specific way: payers and purchasers primarily via *consultation* (i.e., asked to provide written feedback) and experts in law primarily via *participation* (i.e., involved in deliberations and meetings).

When it comes to the methodology used in scoping, the population intervention comparators outcomes (PICO) tool is always used. This tool structures the scoping process, focusing on specifying the research question. Comparators and outcomes are primarily selected based on literature reviews, interviews with health professionals and other relevant experts, and focus groups with a mix of experts (including health professionals and patients). In some cases, relevant outcome measures are selected by surveying relevant stakeholders.

Scoping was also discussed during interviews, confirming that it is often technology-focused, based on literature and expert opinion (see also illustrative fragments from interviews in Table 3 and Supplementary Table 3). At some agencies, stakeholders are consulted about whether they agree with the scope and to raise comments about whether there is anything missing. Interviews on TAVI showed that expectations concerning the health problem (aortic valve stenosis) for which TAVI is held to be a solution, and what the relevant comparators are, are not explicitly questioned during scoping and assumed to be similar to what is claimed by health professionals and/or described in the literature. Consequently, TAVI is only compared with the current standard in clinical practice (SAVR), and alternative interventions (e.g., preventative treatment, drug-based treatment, and so) seem not to be considered. The scoping processes conducted for TAVI are also not reported, only their output is part of the final HTA report (e.g., specifications of objectives or terms of reference for the assessments), or a brief description of input collected from stakeholders during scoping is included in the report (e.g., the NIPH report on TAVI includes an appendix on “user involvement”) (19–22).

**Table 3.** Illustrative fragments from summaries of interviews

Theme	Fragments
Scoping	Not for TAVI for low surgical risk patients, because at the time of the HTA SAVR was considered to be the proper comparator as it was considered the standard of care according to experts in the field. If there would be another relevant comparator, that intervention would already have been tried in the treatment of these patients. And at the time of the HTA, patients at this stage of the disease always received SAVR. We do not question this golden standard in clinical practice. […] Not in the case of TAVI because no other relevant comparator was identified during scoping and this was validated by experts in the field. Additionally, the quantitative and qualitative preferences literature, and engagement with patients, did not identify any other relevant comparators. [Interview #3] As part of the prioritization process, we often provide an initial recommendation about what is required for the topic. For some topics, we will conclude that there is insufficient evidence to support an HTA or that the only information needed is on clinical effectiveness. If it is agreed upon that an HTA is needed and possible, it is discussed with the decision–maker what information is needed for them to make a decision. The outcome of this is the terms of reference for the report, and stakeholders are asked to provide input (e.g., do they miss anything?). [Interview #6]
The use of different types of evidence in assessments of medical devices	No, it’s not a black and white matter. There is some recognition at HTA agencies that real–world data and observational data should be considered in assessments. How I see it is that it renders a methodological inquiry rather than a concern on neutrality and impartiality. The challenge is in integrating these approaches in assessments while simultaneously adhering to the current legal frameworks which are still focused on RCT data. But which types of data are used should depend on the type of questions raised by an assessment. [Interview #2] The requirements on evidence for assessing medical devices should not be different from those for assessing drugs. However, for medical devices the availability of RCTs is often limited, but we always use the highest level of evidence that is available for a given outcome. Therefore, observational data and real–world data can be used to assess medical devices when deemed appropriate. […] The use of observational and/or real–world data for assessing TAVI was part of the discussion before the methodology and literature search was finalized (it was determined during the scoping phase). If observational studies provide information on the same outcomes and for the same follow–up duration as RCTs, and RCTs are of high quality (no risk of bias), RCTs are preferred because they are higher in the hierarchy of evidence. If RCTS are available, observational studies are considered only if they provide additional information to RCTs (i.e., in terms of types and/or duration of outcomes, e.g., longer–term outcomes) or if observational studies are of comparable quality to RCTs. In the case of TAVI, there were two high–quality RCTs available and no information was missed, i.e., there were no observational studies known that could add any relevant information. [Interview #3] What we try to do to address these challenges with medical devices is to make comparisons (e.g., comparing outcomes of interventions using different devices), because that is really important. […] Because, from the perspective of the decision–maker (Ministry of Health) you are focused on the health of the population and the healthcare system, not on a single device. You need information that allows you to compare different technologies to make decisions on that level, to know what you sacrifice if you decide to invest in a particular technology (because resources are limited). [Interviewee #5]
Aspects considered in assessments of medical devices	Quality of life depends on the medical device. We cannot have the quality of life evidence for every medical device. In general, the outcomes depend on the device. […] We look at RCTs, and if not available we use observational studies. If they have reported on quality of life we will include the information in the report, but we do not only focus on it. […] I do think that patient experiences and quality of life is important as a reference for reimbursement decisions, but we do not just focus on patient opinions during the assessment and do not use quality of life as a search key word. [Interview #1] Sometimes decisions are based on things like political expediency, or some other reasons that we cannot capture as part of the evidence base. For example, in the case of orphan drugs, which are not cost–effective, there may be reasons to reimburse them because of care for a group of people who do not have other options. But an HTA struggles to capture that information because it is very hard to do that objectively, although we can highlight it under patient, social and ethical issues. It is not the role of an HTA agency to get everything that is required for the decision, we have to look at the things we can manage objectively. [Interviewee #6] Although the relevance of ethical analysis is acknowledged, in practice, it is mostly not conducted. Important barrier is that the assumption is that it is sufficient that clinicians, health economists, epidemiologists, HTA practitioners, can take ethical aspects into account as part of their analysis. So it is not recognized as a separate domain or analysis step. There is no strong perceived need for an ethicist being explicitly involved in these domains, or a formal integration of an additional ethical analysis. […] It seems to be no one’s concrete responsibility, or all stakeholders (HTA practitioners, decision–makers, etc.) refer to each other. There are different views about what is the appropriate place to address this, some would say that it is the responsibility for political parties or decision–makers. [Interviewee #2]
Stakeholder involvement in assessments of medical devices	In our country, the HTA report is used for reimbursement decisions. When conducting an assessment, we think about the benefits of a technology for society. This means it is important that there is a link with potential benefits for the patients. […] The patient is the most important stakeholder, but not the only one. The perspective and satisfaction of the clinician is also important. For a good use of medical devices, the clinicians and patients are both needed. Both influence the safety and efficacy of medical devices. […] We have to focus on the issues considered relevant by Ministry of Health, both specific issues as a given medical device or wider as pseudo therapies assessments directed to avoid population use them instead of their treatments. [Interview #4] We have been engaging the community and stakeholders in our analysis, but this is hard because people in our country are not used to being involved in these analyses. Therefore, we have been training patients and families about HTA. In addition, the results of an HTA are presented to panels consisting of healthcare professionals that are going to use the device, stakeholders (excluding industry), and the government. These can provide feedback on the results. And a bioethicist and lawyer are usually part of an HTA team, conducting an ethical analysis within the limits of our national law. [Interview #5] Therefore, asking patients whether they can recall a particular experience (prompted by anectodical evidence) may lead to confirmation bias. We cannot base conclusions on anectodical evidence. What we can do is saying that there is some evidence that some patients are unhappy with the intervention, but that it is unclear whether that is a general experience. […] In the case of pharmaceuticals, manufacturers are very clever and know how to involve patients to maximize the chances of a good outcome. For medical devices the manufacturers are not that mature yet, and they involve patients to tell them what is important to them. Only patients can tell you what is important them, and patients are the ones you ultimately want to help. But this needs education, to inform patients about how HTA processes works, and which evidence is required. But it can only be for the good of HTA if patients are more involved and have a better understanding of what is required. But we have to be careful that we do not end up with people that are gaming the system, it is important that the evidence is impartial. And it is important that people think about the greater good. [Interviewee #6]

Interviewees also mentioned that the scope of an assessment is often already pre-determined by legal requirements and/or official HTA guidelines for conducting assessments (see Supplementary Tables 1 and 3).

### Assessment

#### Use of different types of evidence

Participating agencies predominantly use traditional types of studies (e.g., RCT, meta-analysis, systematic review; see Table 4). Also, the use of qualitative research methods is less than 50 percent and confined to obtaining information about patients’ perspectives and experiences to contextualize quantitative evidence, and it has no role as formal evidence in assessments.

**Table 4.** Overview of answers provided to survey questions on evidence considerations in assessments of high-risk medical devices

Question	Answers	Percentage
Which type of studies are primarily considered by your HTA agency when assessing high–risk medical devices? [multiple answers possible] (*n* = 14)	RCT	100%
	Meta–analysis	71%
	Systematic reviews	64%
	Nonrandomized controlled prospective cohort studies	29%
	Primary studies	29%
	Other, please specify: – Comparative study with a control group (*n* = 1) – Other HTA reports (*n* = 1) – Relevant real–world evidence from the healthcare system (if available) (*n* = 1)	21%
Are qualitative research methods (e.g., interviews, focus groups) used by your HTA agency for assessing high–risk medical devices? (*n* = 14)	Yes	43%
	No	57%
For which types of analyses are qualitative research methods considered? [open question] (*n* = 14)	To assess the perspectives and satisfaction of patients regarding the medical device used. For patient perspectives and experiences, caregiver perspectives and experiences, implementation considerations, ethical analysis. Mainly patient and public involvement aspects, e.g., we use available qualitative evidence from literature or primary evidence we collect directly using interviews, focus groups, etc‥ Yes, we evaluated medical device re–manufacturing for the health ministry using a multidimensional approach. For assessment of patients’ perspectives; experts and Qualitative Evidence Synthesis (QES). For signaling inappropriate use and for agenda–setting, not for formal assessments.	
What are the considerations with regard to assessing the quality of evidence when conducting an evaluation of high–risk medical devices? [open question] (*n* = 15)	GRADE (*N* = 6). We consider the internal validity of the studies assessed (i.e., risk of bias) and the applicability to our health system and target population (external validity) in relation with the population (or subgroup of patients with a given baseline characteristics) in which the medical device evaluated is intended to use. Because high–risk medical devices sometimes have ethical issues impeding the conduct of double–blind trials, evidence is sometimes from open–label or without comparator trials, this might affect the quality of evidence. Similar to other technologies (*n* = 2). Assessment of certainty of study results. Study design, population included in the study, comparator, risk of bias, confounding factors. PICO relevance, published in peer–reviewed journals, if necessary we use GRADE.	
Is the quality of evidence interpreted differently for various types of methods (qualitative vs quantitative methods? [open question] (*n* = 15)	“No.” “Yes” (*n* = 2) “Yes, depending on the research questions and studies being included.” “If qualitative is carried out through interviews or focus groups, it may be more open–ended, and many different views and opinions may be collected, or the existing evidence results may be summarized through systematic review, which is less likely understand the actual effect size, and the evidence may come from multiple sources, would lower the quality of the evidence. However, if it is quantitative, the effect size can be provided by statistical methods, but it may also be limited by the quality of the data source and affect the quality of the evidence.” “The certainty and quality of evidence is interpreted according to the specific analysis. There is not the same framework to assess clinical effectiveness and to assess perceived needs from the community because the objectives and the potential outcomes are different.” “Yes. We do not apply/complete formal QA checklists as we operate a rapid review model. But our researchers are highly experienced and apply quality assessment implicitly, drawing out any key issues.” N/A; Qualitative research methods are not (formally) considered in an assessment” (*n* = 6)	

Survey responses and interviews with HTA practitioners show their acknowledgment of challenges involved in collecting data for medical devices, but that they also think the same epistemic principles apply (e.g., evidence hierarchy, risk of bias) and that alternatives like real-world evidence introduce more uncertainty (see Tables 3 and 4, and Supplementary Table 3). What is mentioned several times by HTA practitioners is that they only consider *comparative data*, that is, data that allows you to draw conclusions about the *relative effectiveness* of different health technologies, which is considered important from the viewpoint of the purpose of HTA (to inform decisions on the level of the healthcare system). The main reasons for considering real-world evidence are a) that this could address iterative developments in medical devices (i.e., traditional methods for gathering evidence cannot keep up with this pace of development), and b) to address the context dependency of medical devices (i.e., contextual factors in “real-world” circumstances).

Interviews on TAVI showed (see Table 3 and Supplementary Table 3) that other data types were considered by HTA agencies but not used when assessing safety or comparative clinical effectiveness of medical devices because they were deemed to provide no additional information with respect to available (high-quality) RCT data. The HTA reports on TAVI also show this reliance on RCT data, only one agency (i.e., HIQA) reported findings of registries in their safety assessment, but these were only used as an addition to RCT data. The data from registries was presented only narratively and without any explicit critical appraisal of their quality (besides evaluating the relevance and appropriateness of the included patient populations in registries) (19).

#### Aspects considered in assessment

Aspects primarily considered in assessments of medical devices are *clinical effectiveness* (100 percent), *safety* (93 percent), *costs and economic implications* (79 percent), and quality of life (71 percent), followed by organizational aspects (64 percent), and legal and ethical issues (both 50 percent); see Supplementary Table 4.

Interviewees express a lack of expertise, time, and capacity to consider a broader spectrum of aspects, and that explicit consideration of ethical issues is not always seen as the responsibility of HTA practitioners or is not recognized as requiring explicit attention (see Table 3 and Supplementary Table 3). The inclusion of a broader spectrum of aspects is also limited due to legal frameworks that pre-define a narrower scope for assessments.

For TAVI, Ontario Health assessed a broad range of aspects (clinical effectiveness, safety, cost-effectiveness, budget impact, values and preferences of patients and informal caregivers), and these were integrated in the conclusions and recommendations (20;24;25). Patient preferences were included by reviewing published qualitative and quantitative preferences evidence, and direct engagement of patients with lived experience with TAVI. Ethical issues were not assessed because it was concluded during scoping that there was no need for it. At HIQA, safety, clinical effectiveness, cost-effectiveness, budget impact, and organizational aspects (e.g., impact on healthcare capacity) of TAVI were assessed, whereas ethical issues were only described (with equity as a primary concern) (19). NIPH and HAS assessed the safety, clinical effectiveness, cost-effectiveness, and budget impact of TAVI (21;22).

#### Stakeholder involvement

Stakeholder involvement during assessment is confined to collecting evidence and reviewing its plausibility, and their role in making methodological decisions is limited, see Table 5. Stakeholders involved in all facets of conducting an assessment are patient organizations, providers of care, policymakers, payers/purchasers, and experts in medicine, health economics, epidemiology, ethics, and law. Patients (not represented by an organization), manufacturers, and informal caregivers are involved in collecting evidence, but are almost excluded from making methodological decisions and reviewing evidence.

**Table 5.** Overview of answers provided to survey questions on stakeholder involvement in assessments of medical devices

	Involved in collection of evidence		Involved in making methodological decisions		Involved in reviewing plausibility of evidence reports	
Are stakeholders involved in assessments, at which stage and how?	Yes (*n* = 8) (62%)		Yes (*n* = 3) (23%)		Yes (*n* = 8) (62%)	
	No (*n* = 5) (38%)		No (*n* = 10) (77%)		No (*n* = 5) (38%)	
	**Consultation**	**Participation**	**Consultation**	**Participation**	**Consultation**	**Participation**
Patient’s organization	75%	75%		33%	75%	25%
Providers of care (clinician, nurse, hospital board member, etc.)	63%	63%	33%	67%	63%	38%
Patients with the disease but not yet treated	50%	13%			13%	13%
Patients with the disease and already treated with the comparator	50%	25%			13%	13%
Experts in Medicine	50%	63%		33%	63%	50%
Manufacturers	50%	50%			38%	
Patients treated with the new intervention	38%	13%			13%	13%
Experts in (health) economics	38%	38%	33%	33%	38%	25%
Policymakers	38%	50%	33%	67%	50%	50%
Other	38%	13%	33%	33%	13%	25%
Informal caregivers	25%					
Experts in healthcare administration	25%	38%			13%	
Experts in Epidemiology	25%	25%	33%	33%	38%	38%
Public/(organized) group of citizens	25%	13%			13%	
Experts in Ethics	13%	25%		33%	25%	25%
Experts in Patient and/or Public involvement	13%	13%			13%	
Experts in Bioengineering	13%				13%	13%
Experts in Psychology	13%	13%			25%	
Experts in Law		13%		33%		25%
Payers/purchasers (health insurer, HMO, etc.)		38%	33%	33%	38%	13%
Experts in Sociology					13%	
Experts in Statistics					13%	13%

Interviewees expressed concerns with stakeholder involvement, mentioning potential threats to the impartiality and objectivity of the evidence base, as stakeholders may have vested interests and information provided by them may be skewed to be in favor of certain outcomes. Additionally, interviewees noted that stakeholders have a limited understanding of HTA processes (see Table 3 and Supplementary Table 3). Despite these concerns, interviewees acknowledge the importance of stakeholder involvement, especially for obtaining information on what are relevant outcomes, and to address challenges related to medical devices (e.g., for the appropriate use of medical devices, the engagement of both clinicians and patients is needed; manufacturers can provide technical information about different generations of a device).

Regarding TAVI, stakeholder involvement was limited to a literature review of quantitative and qualitative research into patient preferences, direct engagement of patients (excluding those at low surgical risk), and the inclusion of patient representatives in the Expert Advisory Group. Their direct contributions involved providing feedback to drafts of HTA reports and sharing their experiences (19–21).

## Discussion

Despite the recognized need for changes in HTA methodology for medical devices, HTA agencies still resort to methods developed for assessing drugs and focus on assessing clinical aspects (safety, effectiveness) and cost-effectiveness using quantitative data. The broadening of who is involved (stakeholder involvement), what is assessed (which aspects of health technology), and which information is considered (e.g., real-world evidence, qualitative research), proposed by VALIDATE and other groups of experts in HTA, is not yet fully seen in current practice at HTA agencies (3;8;12). This discrepancy aligns with previous observations in surveys and reviews of guidelines (4;5;9;10). A recently published review of full HTA reports on TAVI for patients at low surgical risk, including the reports discussed in this study, also showed their predominant reliance on traditional RCT data and clinical outcome measures (26). What our findings add to these studies is the understanding that, although HTA practitioners recognize the relevance of other types of evidence and methods, they are committed to existing epistemological principles (e.g., evidence hierarchy, risk of bias) that automatically downgrade non-RCT data, effectively excluding it from having an impact on recommendations as previously observed in a study on real-world data policies for HTA of drugs (27). HTA scholars have also expressed critique on the quality of real-world evidence used in HTAs of high-risk medical devices (28).

Certain practical factors may also explain the reluctance to introducing new methods for assessing medical devices. Both in responses to survey questions and during interviews it became clear that HTA practitioners work under time pressure, must pay attention to the demands of decision-makers, and need to adhere to existing legal frameworks and HTA guidelines, limiting their ability to experiment with new methodology. Therefore, HTA practitioners need a supportive environment (institutional context) that recognizes the importance of changing methodology for assessing medical devices.

In addition to this role of the environment, our interviews with HTA practitioners highlight some normative considerations that also play a role in sustaining the status quo. HTA practitioners frequently expressed concerns about how uncertainties and biases associated with other types of evidence and stakeholders might influence the HTA process, potentially conflicting with the responsibility of HTA to guarantee an impartial (“neutral”, “objective”) synthesis and interpretation of the available evidence. Therefore, the persistent use of traditional methods and evidence hierarchies, and the exclusion of stakeholders in parts of the process, may not only be the result of demands from decision-makers and official frameworks, but also because it is regarded as the best way for ensuring this neutral role of HTA in decision-making. As observed in another interview study, HTA practitioners’ reliance on certain epistemological ideas may originate from ideas about the intrinsic value of HTA itself (13).

Therefore, the adoption of a new methodology for assessing medical devices at HTA agencies requires a discussion within the HTA community about the roles, responsibilities, and goals of HTA, and how to realize them. This includes acknowledging the implicit normative underpinnings of HTA processes and methods. For example, we agree with interviewees that the role and responsibility of HTA is to provide information on the *public value* of health technology, requiring expertise, processes, and methods that ensure collected information is not influenced by interests. However, this does not imply that HTA practitioners need to refrain from making value judgments. Increasingly, HTA agencies and scholars acknowledge that conducting assessments requires making value judgments (29). Although this may be a matter of degree, partly depending on the mandate of the HTA practitioner (e.g., working within a decision-making body or at an academic institute), every assessment requires making value-laden decisions about what are *good* methods and outcome measures to consider in evaluating a health technology (30). Given this recognition of the normativity of HTA, there is room to reflect upon whether current epistemic norms (like the strict adherence to a hierarchy of evidence) are still helpful in fulfilling the role of HTA in decision-making. Methods evolve, offering new ways for obtaining reliable data on the effects of health technology, and HTA guidelines already provide some room to consider diverse outcome measures (31;32). Together with the broader HTA community (those using outcomes of HTA or being impacted by it), HTA practitioners may explore how this new methodology may help in assessing medical devices and improve the relevance of HTA (33).

Future research on the impact of changes in HTA methodology on decision-making, and ideas of decision-makers and stakeholders about evidential requirements for different types of technology, could guide this collaborative rethinking of how new technologies, including medical devices, are assessed (34).

### Strengths and limitations

Although we managed to collect survey responses and conduct interviews with HTA practitioners working at seventeen different agencies, we cannot verify whether we collected all diversity in used methodology and views of HTA practitioners. Future research should try to include more agencies from different regions and interview multiple practitioners per agency. However, we are assured about the validity of our results by the convergence with findings of previous studies on HTA practice for medical devices and interviews with HTA practitioners about their views on appropriate methodology (4;9;10;13;14). By combining surveys and interviews, we have provided an in-depth understanding of *why* certain methodologies are used.

Although we tried to explore websites, published guidelines, and HTA reports of participating agencies, to verify findings, we were sometimes unable to retrieve or understand material because it was not (publicly) available (in English).

## Conclusions

Despite recognizing the need for changes in HTA methodology for medical devices, HTA agencies predominantly use methods developed for assessing drugs. Both practical factors (available capacity, existing legal frameworks, and HTA guidelines) and HTA practitioners’ commitments to principles of evidence-based medicine make adoption of a new methodology difficult. Therefore, the adoption of new methodologies at HTA agencies may require a discussion within the HTA community on the roles, responsibilities, and goals of HTA, and how these can be realized by changes in methodology and institutional context.

## Supporting information

Bloemen and Oortwijn supplementary material 1 Bloemen and Oortwijn supplementary material

Bloemen and Oortwijn supplementary material 2 Bloemen and Oortwijn supplementary material

Bloemen and Oortwijn supplementary material 3 Bloemen and Oortwijn supplementary material

Bloemen and Oortwijn supplementary material 4 Bloemen and Oortwijn supplementary material

## Supplementary material

The supplementary material for this article can be found at http://doi.org/10.1017/S0266462324000254.
