# Supplementary material for: Assessing medical devices: a qualitative study from the validate perspective
Source: Int J Technol Assess Health Care. 2024 Apr 24;40(1):e29. doi: 10.1017/S0266462324000254 (PMC11569912; doi:10.1017/S0266462324000254)
Supplement: Bloemen and Oortwijn supplementary material 1 — Bloemen and Oortwijn supplementary material [file S0266462324000254sup001.docx]

**Supplementary file 1 – survey invitation, survey questions**

**Invitation email survey**

Dear colleague,

We kindly invite you to participate in our study on integrating stakeholder perspectives in health technology assessment (HTA), in which we use the evaluation of high-risk medical devices^[[1]](#footnote-2)^ such as stents or transcatheter aortic valve implantation, as a case study.

The study is inspired by the VALIDATE (VALues In Doing Assessments of health TEchnologies) approach, developed in a recent EU project, of which we (Radboud university medical centre) were the coordinator. VALIDATE has been endorsed by HTAi and EuroScan iHTS and can help HTA practitioners to explicitly address values and stakeholder perspectives in a transparent way. Several HTA agencies have shown interest in applying VALIDATE for which we organize(d) free webinars. For more information: [www.validatehta.eu](http://www.validatehta.eu) and journal articles/handbook: [Downloads – VALIDATE (validatehta.eu)](https://validatehta.eu/downloads/)

The objective of our study is to understand the potential barriers and opportunities for applying the VALIDATE approach in the context of (high-risk) medical devices. Part of the study is an online self-administered survey aiming to 1) map the way in which HTA agencies across the globe incorporate conceptualizations of values and stakeholder perspectives in assessing claims on effects of high-risk medical devices, and 2) identify to what extent scoping (defining the research question) and assessment of these technologies can be enhanced. We kindly ask you to fill out the survey, which has been piloted by a representative of a national HTA agency. The survey can be found via: <https://s.chkmkt.com/?e=298943&h=304DFD1BD828640&l=en>

Please note that all your answers will be treated confidentially: no attribution will be made to specific persons. The results of the study, funded by a non-restricted grant from Edwards Lifesciences, will be submitted for publication in a peer-reviewed journal.

We thank you in advance for your willingness to cooperate and appreciate to receive your reply within 2 weeks.

If you have any question, please let us know.

Very best wishes,

Wija Oortwijn & Bart Bloemen

**Survey**

In this survey, we ask you about the ways in which conceptualizations of values and stakeholder perspectives are incorporated in current practices of A) scoping and B) assessment of high-risk medical devices.

The VALIDATE approach recognizes that judgements on the value of health technology are always closely tied to specific problem definitions of stakeholders. Stakeholders are patients (organizations), informal caregivers, health professionals (clinician, nurse, etc.), academics (health economist, ethicist, epidemiologist, etc.), payers (health insurer etc.), manufacturers, purchasers (hospital board member etc.)) and policy makers.

Stakeholders can all have different problem definitions as these are intertwined with underlying background theory and ethical commitments, which are jointly called *interpretive frames*. An interpretive frame is the set of concepts and theories a stakeholder (implicitly) uses to make sense of concrete situations, and it determines how they judge the situation (as desirable or not) and what questions and concerns it raises for them (https://validatehta.eu/glossary/).

The VALIDATE approach provides strong arguments in favour of an integral role of stakeholder perspectives in HTA and offers concrete suggestions for how these perspectives can be reconstructed and integrated. This opens a space for stakeholder **participation** in which they not only provide input on technology’s value aspects, but on all aspects of conducting and using HTA.

**Consent**

By filling out the survey, you agree for your answers to be analysed and that the results will be presented in anonymous form.

[Yes / No]

**General information**

| What is the name of your organization? |
| --- |
| What is your current position? |
| How many years are you involved in the field of HTA?  < 5 years  5-10 years  >10 years |
| What country or region are you representing? |
| Does your (national/regional) HTA agency evaluate **high-risk medical devices** (i.e. Class IIb, active devices intended to administer and/or remove a medicinal product, and Class III implantable medical devices according to the European Union Regulation on Medical Devices – Regulation (EU) 2017/745)?  Yes, No [**End of survey**]  If yes,  Does the *process* to assess medical devices differ from the assessment of pharmaceuticals at your (national/regional) HTA agency? [Yes/No]   - If Yes, please can you describe what are the differences? [Open question]   Does your (national/regional) HTA agency has a separate unit / department which conducts HTAs for medical devices? [Yes/No]  Please can you specify what definition of medical devices (e.g. in line with current EU-directives) your (national/regional) HTA agency uses? [Open question]  What are the mechanisms for identifying medical devices for HTA in your country/region? [Multiple answers possible]   - Formal request of decision-makers (e.g., Ministry of Health, hospital boards) to HTA agency - Industry submission to HTA agency - Consultation of different stakeholders regarding pre-selected areas - Open consultation of different stakeholders - At the remit of the HTA agency - Horizon scanning systems - Other, please specify….   What criteria are used for the selection of medical devices for assessment? [Multiple answers possible]   - Burden of disease - Economic impact / costs - Potential health benefits - Ethical aspects - Social, cultural and/or legal aspects - Population size - Importance to health care - New indication - Indication extension - Innovativeness - Other, please specify…. |

1. **Scoping**

*The basis of a Health Technology Assessment (HTA) is defining the assessment scope. Scoping includes explicitly defining the objective and research questions of the HTA by a systematic exploration of relevant aspects from multiple perspectives.*

*According to the VALIDATE approach, stakeholders often define a health problem in different ways, giving rise not only to different judgements on the desirability of proposed (technological) solutions, but potentially also to a different set of questions and evidence requirements that would need to be addressed in a specific HTA. It is for this reason that it is advised to start the HTA by exploring which parties may be designated as stakeholder, and how they define the health problem under consideration. Based on that, the objectives of the assessment, the questions that need to be answered, and the evidential requirements, which information needs to be collected, can be specified.*

QUESTIONS:

**Process of scoping**

- Are guidelines / document(s) describing the ***process of scoping*** applicable to the evaluation of high-risk medical devices present in your country/region? [Present and publicly available/ Present but not publicly available/Not Present – **If not present** **go to need for guidance**]

If present:

- - What are the guiding principles of the scoping process described in the guideline(s) / document(s)? [Multiple answers possible]
    - - Overarching goals of HTA agency or health system
      - Guiding principles driven by values such as:
        - Transparency
        - Impartiality
        - Inclusivity
        - Timeliness
        - Consistency
        - Verifiability
        - Efficiency
      - Other, please specify:….
      - Not described
  - What is the **main** focus of the scoping process described in the guideline(s) / document(s)? [Multiple answers possible]
    - - Defining the health technology and the alternative technology(s) against which the health technology under assessment should be compared
      - Defining the relevant research design and evidence requirements
      - Defining to what extent the health problem under study can be addressed (i.e., are non-technological interventions that could be proposed to address the health problem being considered)?
      - Other, please specify……
      - Not described
  - Is the process of stakeholder involvement in scoping described in the guideline(s) / document(s)?
    - - Yes
      - To some extent
      - No [**go to methods**]

If yes/to some extent:

T**he roles and responsibilities of stakeholders involved**

- - - How are stakeholders selected?
      - Open to all (public call)
      - Open to all who qualify (application process)
      - Nominated by relevant interest groups (nomination process)
      - By invitation or appointment (closed procedure)
      - Using a hybrid approach
    - Which input is requested from stakeholders in the scoping process?
      - Stakeholders are explicitly involved in determining the objectives of the assessment
      - The contribution of stakeholders is primarily focused on providing value perspectives and selecting relevant outcomes
      - Background information provided by stakeholders (e.g., experiential knowledge that can help in defining the research question; ideas about the plausibility of different interventions in addressing the health problem; different views on how to define the health problem)
      - Other please specify……
    - The following stakeholders are explicitly involved via **consultation** (structured process to collect feedback among groups of stakeholders on specific decisions via e.g. surveys, interviews, expert panels, patient testimonies) Multiple answers possible]:
      - Patients with the disease but not yet treated
      - Patients with the disease and already treated with the comparator
      - Patients treated with the new intervention
      - Patient's organization
      - Informal caregivers
      - Providers of care (clinician, nurse, hospital board member etc.)
      - Academics/experts in
        - Medicine
        - Law
        - Ethics
        - (Health) Economics
        - Healthcare administration
        - Management science
        - Epidemiology
        - Patient and/or public involvement
        - Bioengineering
        - Political science
        - Sociology
        - Anthropology
        - Psychology
        - Statistics
        - Other, please specify:….
      - Payers / purchasers (health insurer, HMO etc.)
      - Manufacturers
      - Policy makers
      - Public/(organised) group of citizens
      - Other, please specify:…..
    - The following stakeholders are explicitly involved via **participation** (active engagement in deliberations and open exchange on argumentation and evidence) Multiple answers possible]:
      - Patients with the disease but not yet treated
      - Patients with the disease and already treated with the comparator
      - Patients treated with the new intervention
      - Patient's organization
      - Informal caregivers
      - Providers of care (clinician, nurse, hospital board member etc.)
      - Academics/experts in
        - Medicine
        - Law
        - Ethics
        - (Health) Economics
        - Healthcare administration
        - Management science
        - Epidemiology
        - Patient and/or public involvement
        - Bioengineering
        - Political science
        - Sociology
        - Anthropology
        - Psychology
        - Statistics
        - Other, please specify:….
      - Payers / purchasers (health insurer, HMO etc.)
      - Manufacturers
      - Policy makers
      - Public/(organised) group of citizens
      - Other, please specify:…..

**The methods used**

- - Are the methods used in scoping described in the guideline(s) / document(s)?
    - - Yes
      - To some extent
      - No [**go to need for guidance**]

If yes / to some extent

- - - What tool(s) are used for scoping?
      - Population Intervention Comparators Outcomes (PICO) tool
      - Technology Indication Comparison Outcome (TICO) tool
      - Other, please specify:….
    - Are the assumptions concerning working mechanisms that are proposed to justify the potential effectiveness of a health technology (i.e. a hypothesis on how the technology will realize its intended effects) explicitly being questioned in scoping? [Yes/No]
    - What methods are used for selecting relevant outcome measures that need to be considered in an assessment? [Multiple answers possible]:
      - Literature or document review
      - Interviews with health professionals relevant to the disease under study
      - Interviews with patients suffering from the disease under study
      - Interviews with other relevant experts
      - Focus groups with health professionals relevant to the disease under study
      - Focus groups with patients suffering from the disease under study
      - Focus groups with other relevant experts
      - Focus groups with a mix of relevant experts, including health professionals and/or patients
      - Surveys of relevant stakeholders
      - Other, please specify…..
    - What methods are used for selecting comparators (other health technologies, services or interventions) that need to be considered in an assessment? [Multiple answers possible]:
      - Literature or document review
      - Interviews with health professionals relevant to the disease under study
      - Interviews with patients suffering from the disease under study
      - Interviews with other relevant experts
      - Focus groups with health professionals relevant to the disease under study
      - Focus groups with patients suffering from the disease under study
      - Focus groups with other relevant experts
      - Focus groups with a mix of relevant experts, including health professionals and/or patients
      - Surveys of relevant stakeholders
      - Other, please specify…..

**Guidance for scoping**

- With regard to any element listed above that is **not present**: Is there a need for guidance in your country/region regarding a particular element of scoping? [Yes, No]
  - If yes, which one(s), and could you briefly explain why? [Open question]
- Are you aware of any HTA practice that can serve as best practice for scoping? [Yes, No]
  - If yes, please could you indicate which one(s) and provide more information, specifically for:
    - Structuring the process of scoping
    - Involvement of stakeholders (consultation, participation)
    - Methods used

**B) Assessment**

*According to the VALIDATE approach, the assessment process consists of evidence collection, analysis of the quality of the evidence, synthesising the evidence, and reporting the findings and implications. HTA agencies should ideally develop an elaborated HTA protocol to plan this process****,*** *based on the policy question(s) defined during scoping. Several (national/regional/international) guidelines exist for data collection and analysis. Ideally, HTA agencies should present and explain how the collected evidence in evidence reports and standardised evidence summaries for each relevant assessment aspect (defined during scoping) provides an answer to the relevant policy question(s). The reports should be subjected to an independent review and discussed by relevant stakeholders*.

Please answer the following questions by taking **assessing high-risk medical devices** as your reference case.

QUESTIONS:

- Which type of studies are primarily considered by your HTA agency when assessing high-risk medical devices? [Multiple answers possible]:
  - Nonrandomized controlled prospective cohort studies
  - Primary studies
  - RCT
  - Systematic reviews
  - Meta-analysis
  - Other, please specify….
- Which aspects are considered by your HTA agency when assessing high-risk medical devices? [Multiple answers possible]:
  - Clinical effectiveness
  - Quality of life
  - Safety
  - Costs and economic implications
  - Ethical issues
  - Social issues
  - Cultural issues
  - Legal issues
  - Organizational aspects (e.g. improved efficiency)
  - Environmental aspects
  - Wider implications for the patient, relatives, caregivers, and the population (e.g., reducing waiting lists)
  - Other, please specify….
- Do you think that ethical aspects of high-risk medical devices could be assessed according to scientific standards? [Yes/No]
  - Why (not)? [Open question]
  - Which methodologies do you use for assessing ethical aspects? [Multiple answers possible]
    - Specifying norms
    - Coherence analysis (Wide Reflective Equilibrium)
    - Casuistry
    - EUnetHTA Core Model
    - The Socratic (axio­logical) approach
    - Constructive tech­nology assessment
    - Interactive Health Technology Assess­ment
    - Other, please specify.
    - We do not assess ethical aspects
- Are qualitative research methods (e.g. interviews, focus groups) used by your HTA agency for assessing high-risk medical devices? [Yes/**No > go to quality of evidence**]
- If yes, for which type of analyses are qualitative research methods considered? [Open question]
- If yes, how is the validity of the outcomes of qualitative research methods established? [Open question]

**Quality of the evidence**

- What are the considerations with regard to assessing the quality of evidence when conducting an evaluation of high-risk medical devices? [Open question]
- Is the level of evidence different for various types of analyses (e.g., effectiveness, cost-effectiveness, ethics)? [Yes, No].
  - Please could you explain why (not)? [Open question]
- Is the quality of evidence interpreted differently for various types of methods (qualitative vs quantitative methods)? [Yes, No].
  - Please could you explain why (not)? [Open question]
- Does your (national/regional) HTA agency use a tool/template for reporting and summarising the (quality of the) evidence as part of HTA (assessment)? [Yes, No]
  - If yes, could you please provide an example? [Open question]

**Stakeholder involvement:**

- - - - Are stakeholders involved in the collection of evidence?
- **No [go to stakeholders involved in making methodological decisions]**
- Yes, the following stakeholders are explicitly involved via **consultation** (structured process to collect feedback among groups of stakeholders via e.g. surveys, interviews, expert panels, patient testimonies; stakeholders are asked to provide feedback on the evidence collection):
  - - Patients with the disease but not yet treated
    - Patients with the disease and already treated with the comparator
    - Patients treated with the new intervention
    - Patient's organization
    - Informal caregivers
    - Providers of care (clinician, nurse, hospital board member etc.)
    - Academics/experts in
      - Medicine
      - Law
      - Ethics
      - (Health) Economics
      - Healthcare administration
      - Management science
      - Epidemiology
      - Patient and/or public involvement
      - Bioengineering
      - Political science
      - Sociology
      - Anthropology
      - Psychology
      - Statistics
      - Other, please specify:….
    - Payers / purchasers (health insurer, HMO etc.)
    - Manufacturers
    - Policy makers
    - Public/(organised) group of citizens
    - Other, please specify:…..
- Yes, the following stakeholders are explicitly involved via **participation** (active engagement in deliberations and open exchange on argumentation and evidence; stakeholders are actively involved in the collection of evidence):
  - - Patients with the disease but not yet treated
    - Patients with the disease and already treated with the comparator
    - Patients treated with the new intervention
    - Patient's organization
    - Informal caregivers
    - Providers of care (clinician, nurse, hospital board member etc.)
    - Academics/experts in
      - Medicine
      - Law
      - Ethics
      - (Health) Economics
      - Healthcare administration
      - Management science
      - Epidemiology
      - Patient and/or public involvement
      - Bioengineering
      - Political science
      - Sociology
      - Anthropology
      - Psychology
      - Statistics
      - Other, please specify:….
    - Payers / purchasers (health insurer, HMO etc.)
    - Manufacturers
    - Policy makers
    - Public/(organised) group of citizens
      - Other, please specify:…..
- Are stakeholders involved in making methodological decisions in an assessment?
  - **No [go to stakeholders involved in reviewing the plausibility of the evidence reports]**
- Yes, the following stakeholders are explicitly involved via **consultation** (structured process to collect feedback among groups of stakeholders on specific methodological decisions via e.g. surveys, interviews, expert panels):
  - Patients with the disease but not yet treated
  - Patients with the disease and already treated with the comparator
  - Patients treated with the new intervention
  - Patient's organization
  - Informal caregivers
  - Providers of care (clinician, nurse, hospital board member etc.)
  - Academics/experts in
    - Medicine
    - Law
    - Ethics
    - (Health) Economics
    - Healthcare administration
    - Management science
    - Epidemiology
    - Patient and/or public involvement
    - Bioengineering
    - Political science
    - Sociology
    - Anthropology
    - Psychology
    - Statistics
    - Other, please specify:….
  - Payers / purchasers (health insurer, HMO, etc.)
  - Manufacturers
  - Policy makers
  - Public/(organised) group of citizens
  - Other, please specify:…..
    - - Yes, the following stakeholders are explicitly involved via **participation** (active engagement in deliberations and open exchange on methodological decisions):
      - Patients with the disease but not yet treated
      - Patients with the disease and already treated with the comparator
      - Patients treated with the new intervention
      - Patient's organization
      - Informal caregivers
      - Providers of care (clinician, nurse, hospital board member etc.)
      - Academics/experts in
        - Medicine
        - Law
        - Ethics
        - (Health) Economics
        - Healthcare administration
        - Management science
        - Epidemiology
        - Patient and/or public involvement
        - Bioengineering
        - Political science
        - Sociology
        - Anthropology
        - Psychology
        - Statistics
        - Other, please specify:….
      - Payers / purchasers (health insurer, HMO, etc.)
      - Manufacturers
      - Policy makers
      - Public/(organised) group of citizens
      - Other, please specify:…..
- Are stakeholders involved in reviewing the plausibility of the evidence reports?
- No
- Yes, the following stakeholders are explicitly involved via **consultation** (structured process to collect feedback among groups of stakeholders on the plausibility of evidence reports via e.g. surveys, interviews, expert panels):
- Patients with the disease but not yet treated
- Patients with the disease and already treated with the comparator
- Patients treated with the new intervention
- Patient's organization
- Informal caregivers
- Providers of care (clinician, nurse, hospital board member etc.)
- Academics/experts in
  - Medicine
  - Law
  - Ethics
  - (Health) Economics
  - Healthcare administration
  - Management science
  - Epidemiology
  - Patient and/or public involvement
  - Bioengineering
  - Political science
  - Sociology
  - Anthropology
  - Psychology
  - Statistics
  - Other, please specify:….
- Payers / purchasers (health insurer, HMO, etc.)
- Manufacturers
- Policy makers
- Public/(organised) group of citizens
- Other, please specify:…..
- Yes, the following stakeholders are explicitly involved via **participation** (active engagement in deliberations and open exchange on the plausibility of evidence reports):
  - - Patients with the disease but not yet treated
    - Patients with the disease and already treated with the comparator
    - Patients treated with the new intervention
    - Patient's organization
    - Informal caregivers
    - Providers of care (clinician, nurse, hospital board member etc.)
    - Academics/experts in
      - Medicine
      - Law
      - Ethics
      - (Health) Economics
      - Healthcare administration
      - Management science
      - Epidemiology
      - Patient and/or public involvement
      - Bioengineering
      - Political science
      - Sociology
      - Anthropology
      - Psychology
      - Statistics
      - Other, please specify:….
    - Payers / purchasers (health insurer, HMO, etc.)
    - Manufacturers
    - Policy makers
    - Public/(organised) group of citizens
    - Other, please specify:…..

In the following section of questions, we dive a bit deeper into certain assessment aspects that could specifically play a role when assessing **high-risk medical devices.**

Please could you indicate if and how:

- **Contextual factors are taken into account** Context is defined as the conditions and practical circumstances that can be relevant to the uptake and use of an intervention, for example, setting of use (e.g., regional hospital or university hospital) and sociocultural aspects (knowledge, beliefs, conceptions, customs, institutions, and any other capabilities and habits acquired by a group that may influence uptake) [Multiple answers possible]:
  - - By discussing the potential impact of context on the outcomes of the assessment
    - By making methodological choices to account for the potential impact of context on the assessment
    - By quantifying the potential impact of context on outcomes by performing a sensitivity analysis
    - Other, please specify…
    - Not taken into account
- **Implementation issues** are taken into account. Implementation issues refer to the actual delivery of a health technology given national/regional policy measures, protocols and / or funding mechanisms (e.g., tax incentives and reimbursement schemes) that directly or indirectly influence the implementation of the health technology. [[Multiple answers possible]
  - - By discussing the potential impact of implementation issues on the outcomes of the assessment
    - By making methodological choices to account for the potential impact of implementation issues on the assessment
    - By quantifying the potential impact of implementation issues on outcomes by performing a sensitivity analysis
    - Other, please specify…
    - Not taken into account
- **Professionals’ learning effects are taken into account.** Specifically for high-risk devices like transcatheter aortic valve implantation (TAVI), the outcome of an intervention is partly influenced by the experience (and competence) of care providers conducting the procedure. Accumulating experience of care providers may lead to an increase in the effectiveness and/or a decrease in the costs. [Multiple answers possible]
  - - By discussing the potential impact of learning effects on the outcomes of the assessment
    - By making methodological choices to account for the potential impact of learning effects on the assessment
    - By quantifying the potential impact of learning effects on outcomes by performing a sensitivity analysis
    - Other, please specify…
    - Not taken into account
- **Incremental changes are taken into account.** High-risk medical devices like TAVI frequently undergo product modifications, both regarding its technical specifications as its use. This may lead to changes in its efficacy and/or costs. [Multiple answers possible]
  - - By discussing the potential impact of incremental changes on the outcomes of the assessment
    - By making methodological choices to account for the potential impact of incremental changes on the assessment
    - By explicitly demarcating incremental versus disruptive changes
    - By quantifying the potential impact of incremental changes on outcomes by performing a sensitivity analysis
    - Other, please specify…
    - Not taken into account
- **Patient-related factors that influence treatment effects are taken into account.** Patients may respond differently to treatments in terms of nature and magnitude of a beneficial effect, the time of onset, and adverse outcomes. It is therefore important to consider factors that influence treatment effects to determine which treatments work best for whom; making the technology more personalized; and better valuation of outcomes. [ [Multiple answers possible]
  - - By discussing the potential impact of patient-related factors on the outcomes of the assessment
    - By making methodological choices to account for the potential impact of patient-related factors on the outcomes of the assessment
    - By quantifying the potential impact of patient-related factors on the outcomes of the assessment by performing a sensitivity analysis
    - Other, please specify…
    - Not taken into account
- **Patient preferences with regard to treatment outcomes are taken into account.** Patients often have different views on the relative importance of certain treatment outcomes. It is widely acknowledged that understanding patients’ preferences is important for an accurate assessment and appraisal of the impact of a disease on the patient’s quality of life.
  - Not taken into account
  - If Present, how are patient preferences taken into account? [Open question]
- Is there a need for guidance in your country/region regarding a particular element related to **assessment**? [Yes, No]
  - If yes, which one(s), and could you briefly explain why? [Open question]
- With regard to all elements related to the assessment of high-risk medical devices listed in the questions above, are you aware of any HTA practice that can serve as best practice? [Yes, No]
  - If yes, please can you provide your main arguments? [Open question]

**C) Closing**

QUESTIONS:

If you wish to make any comments, questions, or concerns please use the space provided below

May we contact you if we have additional questions? (Yes/No)

If yes, please can you share your email address?

Thank you for taking the time to complete this survey. It is truly appreciated. We will inform you about the results of this survey when the study gets published.

1. According to the European Union Regulation on Medical Devices – Regulation (EU) 2017/745 these include Class IIb, active devices intended to administer and/or remove a medicinal product, and Class III implantable medical devices. [↑](#footnote-ref-2)
