# Supplementary material for: Assessing medical devices: a qualitative study from the validate perspective
Source: Int J Technol Assess Health Care. 2024 Apr 24;40(1):e29. doi: 10.1017/S0266462324000254 (PMC11569912; doi:10.1017/S0266462324000254)
Supplement: Bloemen and Oortwijn supplementary material 2 — Bloemen and Oortwijn supplementary material [file S0266462324000254sup002.docx]

Supplementary file 2 - List of documents used

# Websites of HTA Agencies

- Avalia-t / ACIS: <https://acis.sergas.es/cartafol/1-The-Agency>
- AquAS: <https://aquas.gencat.cat/ca/inici/index.html#googtrans(ca|en)>
- CADTH: <https://www.cadth.ca>
- CDE / HTA: <https://www.cde.org.tw/eng/>
- FOPH: <https://www.bag.admin.ch/bag/en/home/versicherungen/krankenversicherung/krankenversicherung-leistungen-tarife/hta.html>
- G-BA: <https://www.g-ba.de/english/>
- Health Technology Wales: <https://healthtechnology.wales/>
- HIQA: <https://www.hiqa.ie/>
- IECS: <https://www.iecs.org.ar/en/health-technology-assessment-and-health-economics/>
- IETS: <http://www.iets.org.co/>
- IQWiG: <https://www.iqwig.de/en/>
- MaHTAS: <https://www.moh.gov.my/index.php/pages/view/4995?mid=1567>
- NECA: <https://www.neca.re.kr/eng/index.do>
- NIPH: <https://www.fhi.no/en/kn/HTA/>
- SR-NRCHD: <https://www.nrchd.kz/en/for-health-workers/medical-technology-assessment-mta>
- ZIN: <https://www.zorginstituutnederland.nl/over-ons/werkwijzen-en-procedures>

# HTA guidelines, reports, documents

## *HTA guidelines*

- **IQWiG.** General methods. Version 6.1*.* 2022.
- **Malaysian Health Technology Assessment Section (MaHTAS).** Health Technology Assessment Manual. 2021.
- **Ontario Health.** Health Technology Assessments: Methods and Process Guide. 2023.
- **Health Information and Quality Authority.** A Guide to Health Technology Assessment at HIQA. 2016.

## *HTA reports on TAVI*

- **Haute Autorité de Santé.** Commission Nationale D'Évaluation des Dispositifs Medicaux et des Technologies de Santé. EDWARDS SAPIEN 3, bioprothèse valvulaire aortique implantée par voie transfémorale (système COMMANDER) Avis de la CNEDiMTS. 2020.
- **Health Information and Quality Authority.** Health Technology Assessment of transcatheter aortic valve implantation (TAVI) in patients with severe symptomatic aortic stenosis at low and intermediate risk of surgical complications*.* 2019.
- **Norwegian Institute of Public Health**. Transcatheter aortic valve implantation (TAVI) versus surgical aortic valve replacement (SAVR) for patients with severe aortic stenosis and low surgical risk and across surgical risk groups: a health technology assessment. 2021. <https://www.hiqa.ie/sites/default/files/2019-12/TAVI_HTA.pdf>
- **Ontario Health.** Transcatheter aortic valve implantation in patients with severe aortic valve stenosis at low surgical risk: a health technology assessment. *Ont Health Technol Assess Ser [Internet]*. 2020; **20**(14):1-148. <https://www.hqontario.ca/evidence-to-improve-care/health-technology-assessment/reviews-and-recommendations/transcatheter-aortic-valve-implantation-in-patients-with-severe-aortic-valve-stenosis-and-low-surgical-risk>
- **Ontario Health.** Transcatheter aortic valve implantation in patients with severe aortic valve stenosis at low surgical risk: recommendation [Internet]. Toronto (ON): Queen’s Printer for Ontario; 2020 Nov. 5 pp. Available from: <https://www.hqontario.ca/evidence-to-improve-care/health-technology-assessment/reviews-and-recommendations/transcatheter-aortic-valve-implantation-in-patients-with-severe-aortic-valve-stenosis-and-low-surgical-risk>
- **CADTH.** Transcatheter aortic valve implantation for aortic stenosis: a rapid qualitative review. 2019. <https://www.ncbi.nlm.nih.gov/books/NBK549543/>

## *HTA reports*

- **Gallega para la Gestión del Conocimiento en Salud (ACIS), Unidad de Asesoramiento Científico-técnico, Avalia**. Dispositivo de asistencia ventricular izquierda (DAVI) como terapia de destino. 2018.
- **Gallega para la Gestión del Conocimiento en Salud (ACIS), Unidad de Asesoramiento Científico-técnico, Avalia**. Efectividad, seguridad y eficiencia de la técnica de depuración extrahepática MARS® para el tratamiento de la insuficiencia hepatica. 2018.
- **Health Information and Quality Authority.** Health technology assessment of a national deep brain stimulation service in Ireland. Technical report*.* 2012.

## *Documents*

- **Gallega para la Gestión del Conocimiento en Salud (ACIS), Unidad de Asesoramiento Científico-técnico, Avalia**. PriTec Tool: Adaptation for the selection of technologies to be assessed prior entry into the health care benefits basket. 2018.
- **World Health Organization**. *Global atlas of medical devices 2022*. Geneva: World Health Organization; 2022.

# Literature on HTA methodology and processes of selected agencies for assessing medical devices

- **Enzing JJ, Knies S, Boer B, Brouwer WBF.** Broadening the application of health technology assessment in the Netherlands: a worthwhile destination but not an easy ride? *Health Econ Policy Law.* 2021; **10**:111-119.
- **Tsai HY, Huang YW, Chang SY, Huang LY, Lin CJ, Lee PC**. The reimbursement coverage decisions and pricing rules for medical devices in Taiwan. *GMS Health Innov Technol*. 2022; **16**:Doc02.
- **Kinchin I, Walshe V, Normand C, Coast J, Elliott R, Kroll T, et al**. Expanding health technology assessment towards broader value: Ireland as a case study. *Int J Technol Assess Health Care.* 2023; **39** (1), e26, 1-7.
- **Sarimin R, Sabirin J, Ghazali I, Rahim K, Yusof M, et al**. Health Technology Assessment in Malaysia: Past, Present, and Future. *Int J Technol Assess Health Care*. 2019; **35**, 446-451.
