# Supplementary material for: Assessing medical devices: a qualitative study from the validate perspective
Source: Int J Technol Assess Health Care. 2024 Apr 24;40(1):e29. doi: 10.1017/S0266462324000254 (PMC11569912; doi:10.1017/S0266462324000254)
Supplement: Bloemen and Oortwijn supplementary material 3 — Bloemen and Oortwijn supplementary material [file S0266462324000254sup003.docx]

Supplementary file 3 – Interview guide

NOTE: Interviews with the practitioners involved in assessing TAVI were prepared by the lead author by reading and summarizing the published HTA report on TAVI, and visiting the HTA agency website for more information about the institutional context. The interviews with survey respondents were prepared by the lead author by visiting the website of the respective HTA agency to get an overview of the institutional context, tasks, and existing guidelines of the agency, and looking at the responses to the survey questions. Based on these preparations, specific questions for the second part of the interview were formulated to address any gaps in our understanding of the TAVI report and / or existing processes for assessing medical devices in general.

Interview guide for interviews with authors of HTA reports on TAVI

**Material in italics will not be literally read aloud during the interview but is used for further*

*clarification and ensuring that the interviewee is well informed about the purpose of the interview.*

Introduction

*Thank you for agreeing to participate in this interview. We are interviewing you to better understand HTA processes aimed at assessing the value of (high-risk) medical devices, and underlying ideas on methodology in HTA. Based on your experience of being involved in assessing TAVI for treating severe aortic stenosis patients with low surgical risk, we would like to ask you to reflect on general methodological issues in HTA. Our focus will be on the role of normative analysis (i.e. explicating and clarifying what makes a health technology desirable) and the involvement of stakeholders in HTA, which are central elements of the VALIDATE approach towards HTA that we developed (1). This approach can help HTA practitioners to explicitly address values and stakeholder perspectives in a transparent way. The objective of this study is to understand potential barriers and opportunities for applying this approach in the context of medical devices.*

*The interview will contain three parts: (i) questions about your experiences in HTA; (ii) questions about the institutional background and context of your HTA report on TAVI; (iii) questions about your views on the role and methodology of HTA for medical devices.*

*The interview should take approximately one hour. With your permission, I would like to audio record the interview. All responses will be kept confidential, which means that your de-identified interview responses will be only shared with research team members and we will ensure that any information that we include in our publications will not identify you as the respondent. You may decline to answer any question or stop the interview at any time and for any reason.*

Interview

*Please note that this guide only represents the main themes to be discussed with the participants and as such does not include the various prompts that may also be used (examples given for each question). Non-leading and general prompts will also be used, such as “Can you please tell me a little bit more about that?” and “How does that look like for you”.*

**Part 1. Position and experience in HTA**

Before we begin, it would be nice if you could tell me a little bit about yourself.

- Can you tell us something about your professional background?
- What is your current position?
- How many years of experience do you have in HTA?
- In which assessments of medical devices where you involved previously?
- What was your role in the HTA of TAVI?

**Part 2. Institutional practices and the context of the HTA report on TAVI**

*To understand the context of the published HTA report on TAVI, we would like to ask some questions about your institution (for which you conducted the HTA on TAVI). In addition, we ask questions on the choices being made while conducting the assessment on TAVI, and the impact of the report on actual decision-making, which are both aspects of the assessment that may not have been described in the HTA report. This part of the interview may also contain some specific questions asking for clarifications of aspects of the published HTA report on TAVI.*

| Main question | Prompts | Rationale, sources |
| --- | --- | --- |
| 1. What is the role of your HTA agency in the decision-making process on medical devices in your country? | *Does your agency make policy recommendations or is it only involved in conducting assessments? How are the HTA reports that your agency publishes used in decision-making on medical devices? What is the formal status of your conclusions and/or recommendations (e.g. are assessments directly informing coverage decision-making?)*  *How is your agency related to decision-making bodies at regional or federal levels?*  *Does your agency have a separate unit or department which conducts HTAs for medical devices?* | To understand the context of the HTA report on TAVI. (2) |
| 2. Who initiated the HTA on TAVI? | *What was the reason for conducting this assessment on TAVI?*  *How, and who (e.g. the developers, government, healthcare professionals), do you decide which devices to assess? Priority setting?*  *Who was involved in determining the scope (e.g. objectives, research questions, criteria / outcome measures, comparators) of the assessment? Did you use any specific tool for scoping (e.g. PICO, TICO, other) or was the scope determined by an official guideline or legal framework? Which substantial and procedural values does your agency promote? How is that reflected in the formal criteria that you used in the assessment? Are the outcomes of scoping published?* | To understand the context of the HTA report on TAVI, and the initial reasons for conducting the assessment. |
| 3. How where the main findings in your report on TAVI reached? | *Why did you consider these types of evidence (e.g. cohort studies, primary studies, RCT, systematic reviews, meta-analysis, qualitative research, other…)? Where other types of information considered? Did you miss any information that would have helped in the assessment process?*  *How and why (not) were stakeholders involved in the generation of your HTA report on TAVI? Do you think that they should have been involved? Are there gaps in knowledge on effects of TAVI that stakeholders could help resolving?*  *Was there any ethical review or analyses conducted? Why?*  *How were different aspects / analyses (e.g. safety, clinical effectiveness etc.) integrated into a conclusion or recommendation? Any ranking or knock-out criteria?*  *Specific questions on the decisions made in the assessment (normative decisions)…* | To understand the (hidden / implicit) decisions being made in conducting the assessment and writing the HTA report on TAVI. |
| 4. What was the impact of your HTA report on the use of TAVI in your country? | *How were the findings of your HTA report used by decision makers? To what extent did it determine the decisions made?*  *How is TAVI currently used in your country? To what extend did the findings of your HTA report change the way TAVI is used in your country?*  *Does your agency have any role in monitoring of long-term effects of TAVI?* | To understand the impact of the assessment on the actual implementation of TAVI. |

**Part 3. Views on the role and methodology of HTA**

*This part of the interview is about your personal views on the role and methodology of HTA, with a focus on HTA for medical devices. The goal is to understand how views on the appropriate role, methodology and evidence required for HTA are associated with underlying values and norms. This may help in understanding HTA practitioners' views on what HTA should do, and whether, and how, they see a role for normative analysis and stakeholder involvement in HTA (an approach towards HTA like VALIDATE). Therefore, although your views are of course shaped by your experience and the position of your HTA agency, we like to hear your personal views (as a professional) to understand how guidelines and methodologies are used and interpreted in the practice of HTA and whether there is room for any changes.*

*Although the questions are of a more general nature, we ask you to use your experiences with assessing medical devices in answering these questions.*

| Main question | Prompts | Rationale, sources |
| --- | --- | --- |
| 5. What is – according to you - the contribution of an HTA agency to society? | *Would you say that the goal of HTA is primarily to inform decision-making processes and increase the quality of resulting decisions, or to empower citizens and other stakeholders by including them into decision-making processes and enhance their democratic legitimacy, or to facilitate and steer innovation to achieve better societal (or patient) outcomes?*  *Should HTA agencies contribute to developing a shared view on what makes a health technology desirable?* | HTA agency – membership criteria of INAHTA  Normativity *of* HTA: different ideas about the role of HTA; these ideas may influence decisions in preparing and conducting an assessment; (Based on discussion in literature on purpose of (H)TA) (3-9);  VALIDATE states that HTA should aim to help stakeholders collaboratively reaching a judgment regarding the value of a health technology (1) |
| 6. What should be the role of the individual HTA practitioner in conducting assessments and informing decision-making processes? | *It has been mentioned in literature that HTA practitioners adopt many roles. Which role description would best suit your work in HTA: the neutral and distant advisor, a facilitator of deliberation, a governance facilitator, or an agenda-setter?*  *Would you like adopting other roles? Why?*  *In literature, some HTA practitioners refer to a commitment to neutrality, implying that the role of an HTA practitioner should be restricted to providing objective information (information that is free from values, biases, emotions). Do you think that, in order to fulfil its role in society, an HTA practitioner should provide objective information? Or does an HTA practitioner also make value judgments (* ‘Value judgment’: there is flexibility from a scientific perspective – more than one methodology or approach is possible, having potential social or ethical consequences following a decision)?*  *Are there any situations in which you feel that you have to make value judgments as HTA practitioner?*  *Do you think that addressing value judgments rules out or threatens the possibility of providing good evidence?* | Normativity *of* HTA, moral commitments; To understand how HTA practitioners view their own role and expertise in the decision-making process, different roles are described in literature (10); To understand whether HTA practitioners are committed to the ideal of providing objective information, and whether they think that value judgments should be avoided as much as possible (which may impede the integration of normative analysis as proposed by the VALIDATE approach) (4; 10-12) |
| 7. What are the most important outcomes to include when assessing medical devices? | *In literature it is mentioned that medical devices often aim at improving outcomes beyond health-related quality of life. Do you think that HTA agencies should restrict their work to assessing effectiveness (in terms of health-related quality of life) and costs, or could also assess other outcomes (e.g. well-being, fairness, autonomy)?*  *Do you think it is possible to assess outcomes like well-being, fairness, autonomy? Why and how?*  *Are there aspects of medical devices that could be addressed by qualitative research? Would you say that this is appropriate evidence?*  *Are there outcome measures that, according to you, are more or less ‘objective’? Does that matter with respect to their relevance and appropriateness for HTA?*  *Should stakeholders be involved in selecting and developing outcome measures?* | Moral and epistemological commitments; Discussion on relevant outcome measures for HTA (8; 13); in particular for medical devices: often aimed at improving outcomes beyond health-related quality of life? (14; 15); Retrieve views on what should be the focus in HTA (e.g. prioritizing on cost-effectiveness or on fairness; desirability or affordability) (8); Retrieve views on whether the assessment of medical devices demands other evidential requirements than pharmaceuticals (14-16);  The VALIDATE approach states that outcomes should be selected in collaboration with stakeholders, and derive its relevance from ethical commitments (1) |
| 8. How can stakeholders contribute to assessments of medical devices? | *In literature there is discussion on the aim of stakeholder involvement of HTA, with purposes ranging from legitimizing decisions to collecting information. According to you, what should be the primary aim of stakeholder involvement (e.g. promote fairness, enhance public support for decisions, help ensure that decisions reflect public values, gather valuable insights and evidence, help methodologies to evolve)?*  *At which phases of the process should stakeholders be included (e.g. scoping, data collection, assessment, recommendations)?*  *What should be the level of involvement: consultation (i.e. approaches to obtain the perspectives of stakeholders, e.g. questionnaires, surveys, interviews, discussion groups), collaboration (i.e. approaches in which stakeholders help with carrying out of a project, e.g. work groups, expert committees), or co-construction (i.e. simultaneous engagement of stakeholders and professionals, based on the complementarity of each’s expertise and experiential knowledge, e.g. joint expert committees)?*  *Do you think that stakeholders can be a reliable source of information in HTA, i.e. should their experiences (captured by stories, testimonials, interviews etc.) used as a formal type of evidence in HTA? Do stakeholders have the requisite knowledge and skills to provide information into the HTA process?*  *There have been cases where there is a conflict between evidence and what clinical expertise and patients’ experiences say about the effectiveness of a health technology. When experiences of patients contradict biomedical evidence, what should you conclude?*  *Based on the guidelines or methods handbook that your institution uses, are you allowed to include forms of information provided by patients or other stakeholders?* | Moral and epistemological commitments;  - To understand the role that stakeholders could play in HTA of medical devices (1; 15; 17)  - To understand the views of HTA practitioners on stakeholder involvement, especially on whether they can have epistemic contributions, given that ideas on appropriate / valid evidence are mentioned as one of the barriers to stakeholder involvement (11; 18-21) and debated in court (22)  - To understand the views of HTA practitioners on the desirability of stakeholder involvement, and its relation with the goals of HTA (23; 24) |
| 9. Do you think that real-world evidence could be used in assessments of medical devices? | *In literature it is mentioned that difficulties in setting up RCTs for medical devices, and certain characteristics of these technologies (iterative development, context-dependency, learning curve), requires the use of real-world evidence in their assessment? What is your view on this?*  *Another associated challenge with the evidence base for medical devices is uncertainty. Do you think that the uncertainties in evidence on the clinical effectiveness of medical devices are different from drugs (or other types of health technologies)? Would this imply setting other evidential requirements for medical devices?*  *If the evidence on the effects of a medical device is inconclusive, or if there are questions currently not being addressed in literature, should an HTA agency postpone drawing any conclusions and ask for additional research being done? Could an HTA agency conduct this research to address evidence gaps?*  *Could coverage with evidence development (CED) schemes be used for medical devices? Would you recommend such a scheme for high-risk devices like TAVI?* | Epistemological commitments; in literature it is often mentioned that medical devices pose specific challenges to current approaches in collecting evidence on effectiveness and cost-effectiveness (iterative development, importance of context and user, less stringent market entry conditions, infeasibility of RCTs) (14; 16; 25-27); Retrieve views on whether the assessment of medical devices demands other evidential requirements than pharmaceuticals, such as the use of real-world evidence (14-16; 28), or new policies like coverage with evidence development (29); The VALIDATE approach states that judgments on the relevance of types of evidence in HTA are always derived from underlying ideas about desirable and plausible characteristics of health technology (1) |
| 10. Should HTA agencies, in determining their agenda, be more focused on issues raised by specific technologies or on addressing general problems in healthcare? | *Should HTA respond to questions being raised by the introduction and use of specific health technologies, or should it focus on identifying and addressing general problems in healthcare? Should it be actively involved in identifying and defining problems in healthcare?*  *Should an HTA agency question the assumptions concerning the working mechanism of a technology?*  *Should alternative policy options and interventions (e.g. social care, preventative measures etc.) for addressing health problems be taken into account in an assessment?*  *Is the nature of medical devices different from other types of health technology? Does the nature of different types of health technology need to be taken into account in HTA methodology or should HTA be ‘technology agnostic’?* | Ontological commitments; retrieve underlying views on causes of a healthcare problem, nature of technology, should HTA be technology or problem driven? The VALIDATE approach states that health technologies are a sort of policy solutions proposed to address particular health problems, and HTH should always critically assess the underlying problem definition (1; 8; 30; 31) |

Closure

Finally, do you have any questions for me? Is there anything else that you would like to comment on that I haven’t already asked you about?

[Stop audio recording]

The results of the analysis of the interviews will be submitted for publication in a peer-reviewed journal, and you will be informed about this. We may ask you to clarify certain answers and / or provide comments to our analysis.

Another part of our study is a survey on procedures and methodology for medical devices in general. Did you see the invitation? Would you, or colleagues, be willing to fill out the survey?

Do you know a colleague that we should invite for an interview to gain more information about HTA of medical devices?

If you have any remaining questions or comments, feel free to contact us.

Thank you for your participation and help!

References

1. van der Wilt G.J., Bloemen B., Grin J., et al. Integrating Empirical Analysis and Normative Inquiry in Health Technology Assessment: The Values in Doing Assessments of Health Technologies Approach. International Journal of Technology Assessment in Health Care, 2022, 38(1).

2. Organization W.H. Global atlas of medical devices. 2017.

3. Van Oudheusden M., Delvenne P. & Lucivero F. Making the invisible visible. TATuP Zeitschrift für Technikfolgenabschätzung in Theorie und Praxis, 2019, 28(1), 21-26.

4. Torgersen H. Three myths of neutrality in TA - How different forms of TA imply different understandings of neutrality. Technological Forecasting and Social Change, 2019, 139, 57-63.

5. Gagnon H., Legault G.A., Bellemare C.A., et al. How does HTA addresses current social expectations? An international survey. Int J Technol Assess Health Care, 2020, 37, e9.

6. Culyer A. & Husereau D. Redefining Health Technology Assessment: A Comment on "The New Definition of Health Technology Assessment: A Milestone in International Collaboration". Int J Technol Assess Health Care, 2022, 38(1), e54.

7. O'Rourke B., Oortwijn W. & Schuller T. Response to redefining health technology assessment: a comment on "the new definition of health technology assessment: a milestone in international collaboration". Int J Technol Assess Health Care, 2022, 38(1), e55.

8. Lehoux P. The problem of health technology. New York: Routledge, 2006.

9. Goetghebeur M.M., Wagner M., Samaha D., et al. Exploring Values of Health Technology Assessment Agencies Using Reflective Multicriteria and Rare Disease Case. Int J Technol Assess Health Care, 2017, 33(4), 504-20.

10. Bauer A. & Kastenhofer K. Policy advice in technology assessment: Shifting roles, principles and boundaries. Technological Forecasting and Social Change, 2019, 139, 32-41.

11. Ducey A., Ross S., Pott T. & Thompson C. The moral economy of health technology assessment: an empirical qualitative study. Evidence & Policy: A Journal of Research, Debate and Practice, 2017, 13(1), 7-28.

12. Harvard S. & Werker G.R. Health Economists on Involving Patients in Modeling: Potential Benefits, Harms, and Variables of Interest. Pharmacoeconomics, 2021, 39(7), 823-33.

13. Brazier J.E., Rowen D., Lloyd A. & Karimi M. Future Directions in Valuing Benefits for Estimating QALYs: Is Time Up for the EQ-5D? Value Health, 2019, 22(1), 62-68.

14. Enzing J.J., Knies S., Boer B. & Brouwer W.B.F. Broadening the application of health technology assessment in the Netherlands: a worthwhile destination but not an easy ride? Health Econ Policy Law, 2020, 1-17.

15. Fuchs S., Olberg B., Panteli D., Perleth M. & Busse R. HTA of medical devices: Challenges and ideas for the future from a European perspective. Health Policy, 2017, 121(3), 215-29.

16. Enzing J.J., Vijgen S., Knies S., Boer B. & Brouwer W.B.F. Do economic evaluations of TAVI deal with learning effects, innovation, and context dependency? A review. Health Policy and Technology, 2021, 10(1), 111-19.

17. Pomey M.P., Brouillard P., Ganache I., et al. Co-construction of health technology assessment recommendations with patients: An example with cardiac defibrillator replacement. Health Expect, 2020, 23(1), 182-92.

18. Steffensen M.B., Matzen C.L. & Wadmann S. Patient participation in priority setting: Co-existing participant roles. Soc Sci Med, 2022, 294, 114713.

19. Staniszewska S. & Soderholm Werko S. Mind the evidence gap: the use of patient-based evidence to create "complete HTA" in the twenty-first century. Int J Technol Assess Health Care, 2021, 37, e46.

20. Bidonde J., Vanstone M., Schwartz L. & Abelson J. An institutional ethnographic analysis of public and patient engagement activities at a national health technology assessment agency. Int J Technol Assess Health Care, 2021, 37, e37.

21. Cleemput I., Dauvrin M., Kohn L., et al. Developing an agency's position with respect to patient involvement in health technology assessment: the importance of the organizational culture. Int J Technol Assess Health Care, 2020, 36(6), 569-78.

22. Moes F., Houwaart E., Delnoij D. & Horstman K. Contested evidence: a Dutch reimbursement decision taken to court. Health Econ Policy Law, 2017, 12(3), 325-44.

23. Wale J.L., Chandler D., Collyar D., et al. Can We Afford to Exclude Patients Throughout Health Technology Assessment? Front Med Technol, 2021, 3, 796344.

24. Moes F., Houwaart E., Delnoij D. & Horstman K. Questions regarding 'epistemic injustice' in knowledge-intensive policymaking: Two examples from Dutch health insurance policy. Soc Sci Med, 2020, 245, 112674.

25. Hulstaert F., Pouppez C., Primus-de Jong C., Harkin K. & Neyt M. Evidence gaps for drugs and medical devices at market entry in Europe and potential solutions. KCE Reports. Brussels: Belgian Health Care Knowledge Centre (KCE); 2021.

26. Ming J., He Y., Yang Y., et al. Health technology assessment of medical devices: current landscape, challenges, and a way forward. Cost Eff Resour Alloc, 2022, 20(1), 54.

27. Tarricone R., Torbica A. & Drummond M. Challenges in the Assessment of Medical Devices: The MedtecHTA Project. Health Econ, 2017, 26 Suppl 1, 5-12.

28. Pongiglione B., Torbica A., Blommestein H., et al. Do existing real-world data sources generate suitable evidence for the HTA of medical devices in Europe? Mapping and critical appraisal. Int J Technol Assess Health Care, 2021, 37(1), e62.

29. Federici C., Reckers-Droog V., Ciani O., et al. Coverage with evidence development schemes for medical devices in Europe: characteristics and challenges. Eur J Health Econ, 2021, 22(8), 1253-73.

30. Kluytmans A., Tummers M., van der Wilt G.J. & Grutters J. Early Assessment of Proof-of-Problem to Guide Health Innovation. Value Health, 2019, 22(5), 601-06.

31. Giacomini M., Winsor S. & Abelson J. Ethics in Health Technology Assessment: Understanding Health Technologies as Policies. Healthcare Management Forum, 2013, 26(2), 72-76.

Interview guide for interviews with survey respondents

**Material in italics will not be literally read aloud during the interview but is used for further*

*clarification and ensuring that the interviewee is well informed about the purpose of the interview.*

# Introduction

*Thank you for agreeing to participate in this interview. We are interviewing you to better understand HTA processes aimed at assessing the value of (high-risk) medical devices, and underlying ideas on methodology in HTA. Based on your experience of being involved in assessing medical devices, we would like to ask you to reflect on general methodological issues in HTA. Our focus will be on the role of normative analysis (i.e. explicating and clarifying what makes a health technology desirable) and the involvement of stakeholders in HTA, which are central elements of the VALIDATE approach towards HTA that we developed (1). This approach can help HTA practitioners to explicitly address values and stakeholder perspectives in a transparent way. The objective of this study is to understand potential barriers and opportunities for applying this approach in the context of medical devices.*

*The interview will contain three parts: (i) questions about your experiences in HTA; (ii) questions about your answers provided to our survey on HTA for medical devices; (iii) questions about your views on the role and methodology of HTA for medical devices.*

*The interview should take approximately one hour. With your permission, I would like to audio record the interview. All responses will be kept confidential, which means that your de-identified interview responses will be only shared with research team members and we will ensure that any information that we include in our publications will not identify you as the respondent. You may decline to answer any question or stop the interview at any time and for any reason.*

# Interview

*Please note that this guide only represents the main themes to be discussed with the participants and as such does not include the various prompts that may also be used (examples given for each question). Non-leading and general prompts will also be used, such as “Can you please tell me a little bit more about that?” and “How does that look like for you”.*

**Part 1. Position and experience in HTA**

Before we begin, it would be nice if you could tell me a little bit about yourself.

- Can you tell us something about your professional background?
- What is your current position?
- How many years of experience do you have in HTA?
- In which assessments of medical devices where you involved previously?
- What was your contribution to these assessments?

**Part 2. Institutional practices and methods for HTA of medical devices**

*To understand the institutional landscape and current methodology for HTA of medical devices, we have send out a survey to HTA practitioners to ask them how HTA of medical devices is conducted at their agency. In this part of the interview we want to raise some in-depth questions to understand answers given and check responses. In addition, this part of the interview may also contain some specific questions asking for clarifications of answers given and methodologies used at your institute.*

| Main question | Prompts | Rationale, sources |
| --- | --- | --- |
| 1. What is the role of your HTA agency in the decision-making process on medical devices in your country? | *Does your agency make policy recommendations or is it only involved in conducting assessments? How are the HTA reports that your agency publishes used in decision-making on medical devices? What is the formal status of your conclusions and/or recommendations (e.g. are assessments directly informing coverage decision-making?)*  *How is your agency related to decision-making bodies at regional or federal levels?*  *Does, and how, the process of decision-making on medical devices differ from the process for pharmaceuticals? Why?* | To understand the institutional context. |
| 2. How are HTAs of medical devices initiated? How is the scope determined? | *Specific questions to clarify answers given to survey questions on selecting medical devices for assessment and determining the scope…* | To understand the scoping process. |
| 3. Which methodology do you use for assessing medical devices? | *Specific questions to clarify answers given to survey questions on assessment of medical devices…* | To understand the methodological decisions being made in conducting assessments of medical devices. |
| 4. What is the impact of your HTA agency on the use of medical devices in your country? | *How are findings of your HTA reports used by decision makers? To what extent do they determine the decisions being made?*  *How are medical devices currently introduced and used in your country? To what extend does the work of your HTA agency influence this use of medical devices?*  *Does your agency monitor or evaluate the impact of its work on the use of medical devices? If so, how? Or why not?* | To understand the impact of assessments on the actual implementation of medical devices, and how HTA practitioners think about the relation between HTA findings and actual decisions. |

**Part 3. Views on the role and methodology of HTA**

*This part of the interview is about your personal views on the role and methodology of HTA, with a focus on HTA for medical devices. The goal is to understand how views on the appropriate role, methodology and evidence required for HTA are associated with underlying values and norms. This may help in understanding HTA practitioners' views on what HTA should do, and whether, and how, they see a role for normative analysis and stakeholder involvement in HTA (an approach towards HTA like VALIDATE). Therefore, although your views are of course shaped by your experience and the position of your HTA agency, we like to hear your personal views (as a professional) to understand how guidelines and methodologies are used and interpreted in the practice of HTA and whether there is room for any changes.*

*Although the questions are of a more general nature, we ask you to use your experiences with assessing medical devices in answering these questions.*

| Main question | Prompts | Rationale, sources |
| --- | --- | --- |
| 5. What is – according to you - the contribution of an HTA agency to society? | *What is the main societal challenge concerning the introduction and use of health technology? To which policy and / or decision problems does it lead? Why do we need HTA to address this challenge?*  *Would you say that the goal of HTA is primarily to inform decision-making processes and increase the quality of resulting decisions, or to empower citizens and other stakeholders by including them into decision-making processes and enhance their democratic legitimacy, or to facilitate and steer innovation to achieve better societal (or patient) outcomes?*  *Do HTA agencies primarily serve the decision-maker or society? Should they question the ideas of decision-makers on what are relevant topics and questions that need an assessment?*  *Should HTA agencies contribute to developing a shared view on what makes a health technology desirable?* | To explore different ideas about the purpose and role of HTA; these ideas may influence decisions in preparing and conducting an assessment (2-8);  VALIDATE states that HTA should aim to help stakeholders collaboratively reaching a judgment regarding the value of a health technology (1) |
| 6. What should be the role of the *individual* HTA practitioner in conducting assessments and informing decision-making processes? | *It has been mentioned in literature that HTA practitioners adopt many roles. Which role description would best suit your work in HTA: the neutral and distant advisor, a facilitator of deliberation, a governance facilitator, or an agenda-setter?*  *Would you like adopting other roles? Why?*  *In literature, some HTA practitioners refer to a commitment to neutrality, implying that the role of an HTA practitioner should be restricted to providing objective information (information that is free from values, biases, emotions). Do you think that, in order to fulfil its role in society, an HTA practitioner should provide objective information? Or does an HTA practitioner also make value judgments (* ‘Value judgment’: there is flexibility from a scientific perspective – more than one methodology or approach is possible, having potential social or ethical consequences following a decision)?*  *Are there any situations in which you feel that you have to make value judgments as HTA practitioner?*  *Do you think that addressing value judgments rules out or threatens the possibility of providing good evidence?* | Normativity *of* HTA, moral commitments; To understand how HTA practitioners view their own role and expertise in the decision-making process, different roles are described in literature (9); To understand whether HTA practitioners are committed to the ideal of providing objective information, and whether they think that value judgments should be avoided as much as possible (which may impede the integration of normative analysis as proposed by the VALIDATE approach) (3; 9-11) |
| 7. What are the most important outcomes to include when assessing medical devices? | *In literature it is mentioned that medical devices often aim at improving outcomes beyond health-related quality of life. Do you think that HTA agencies should restrict their work to assessing effectiveness (in terms of health-related quality of life) and costs, or could they also assess other outcomes (e.g. well-being, fairness, autonomy)? Do you think it is possible to assess outcomes like well-being, fairness, autonomy? Why and how?*  *Are there aspects of medical devices that could be addressed by qualitative research? Would you say that this is appropriate evidence?*  *Are there outcome measures that, according to you, are more or less ‘objective’? Does that matter with respect to their relevance and appropriateness for HTA?*  *Should stakeholders be involved in selecting and developing outcome measures?* | Moral and epistemological commitments; Discussion on relevant outcome measures for HTA (7; 12); in particular for medical devices: often aimed at improving outcomes beyond health-related quality of life? (13; 14); Retrieve views on what should be the focus in HTA (e.g. prioritizing on cost-effectiveness or on fairness; desirability or affordability) (7); Retrieve views on whether the assessment of medical devices demands other evidential requirements than pharmaceuticals (13-15);  The VALIDATE approach states that outcomes should be selected in collaboration with stakeholders, and derive its relevance from ethical commitments (1) |
| 8. How can stakeholders contribute to assessments of medical devices? | *In literature there is discussion on the aim of stakeholder involvement of HTA, with purposes ranging from legitimizing decisions to collecting information. According to you, what should be the primary aim of stakeholder involvement (e.g. promote fairness, enhance public support for decisions, help ensure that decisions reflect public values, gather valuable insights and evidence, help methodologies to evolve)?*  *At which phases of the process should stakeholders be included (e.g. scoping, data collection, assessment, recommendations)?*  *What should be the level of involvement: consultation (i.e. approaches to obtain the perspectives of stakeholders, e.g. questionnaires, surveys, interviews, discussion groups), collaboration (i.e. approaches in which stakeholders help with carrying out of a project, e.g. work groups, expert committees), or co-construction (i.e. simultaneous engagement of stakeholders and professionals, based on the complementarity of each’s expertise and experiential knowledge, e.g. joint expert committees)?*  *Do you think that stakeholders can be a reliable source of information in HTA, i.e. should their experiences (captured by stories, testimonials, interviews etc.) used as a formal type of evidence in HTA? Do stakeholders have the requisite knowledge and skills to provide information into the HTA process?*  *There have been cases where there is a conflict between evidence and what clinical expertise and patients’ experiences say about the effectiveness of a health technology. When experiences of patients contradict evidence, what should you conclude?* | Moral and epistemological commitments;  - To understand the role that stakeholders could play in HTA of medical devices (1; 14; 16)  - To understand the views of HTA practitioners on stakeholder involvement, especially on whether they can have epistemic contributions, given that ideas on appropriate / valid evidence are mentioned as one of the barriers to stakeholder involvement (10; 17-20) and debated in court (21)  - To understand the views of HTA practitioners on the desirability of stakeholder involvement, and its relation with the goals of HTA (22; 23) |
| 9. Do you think that real-world evidence should be used in assessments of medical devices? | *In literature it is mentioned that difficulties in setting up RCTs for medical devices, and certain characteristics of these technologies (iterative development, context-dependency, learning curve), requires the use of real-world evidence in their assessment? What is your view on this?*  *Another associated challenge with the evidence base for medical devices is uncertainty. Do you think that the uncertainties in evidence on the clinical effectiveness of medical devices are different from drugs (or other types of health technologies)? Would this imply setting other evidential requirements for medical devices?*  *If the evidence on the effects of a medical device is inconclusive, or if there are questions currently not being addressed in literature, should an HTA agency postpone drawing any conclusions and ask for additional research being done? Could an HTA agency conduct this research to address evidence gaps?* | Epistemological commitments; in literature it is often mentioned that medical devices pose specific challenges to current approaches in collecting evidence on effectiveness and cost-effectiveness (iterative development, importance of context and user, less stringent market entry conditions, infeasibility of RCTs) (13; 15; 24-26); Retrieve views on whether the assessment of medical devices demands other evidential requirements than pharmaceuticals, such as the use of real-world evidence (13-15; 27); The VALIDATE approach states that judgments on the relevance of types of evidence in HTA are always derived from underlying ideas about desirable and plausible characteristics of health technology (1) |
| 10. Should HTA agencies, in determining their agenda, be more focused on issues raised by specific technologies or on addressing general problems in healthcare? | *Should HTA agencies respond to questions being raised by the introduction and use of specific health technologies, or should it focus on identifying and addressing general problems in healthcare? Should it be actively involved in identifying and defining problems in healthcare (i.e. facilitating deliberations to reach a common problem definition / vision)?*  *Should alternative policy options and interventions (e.g. social care, preventative measures etc.) for addressing health problems be taken into account in an assessment?*  *Should an HTA agency question the assumptions concerning the working mechanism of a technology?*  *Is the nature of medical devices different from other types of health technology? Does the nature of different types of health technology need to be taken into account in HTA methodology or should HTA be ‘technology agnostic’?* | Ontological commitments; retrieve underlying views on causes of a healthcare problem, nature of technology, should HTA be technology or problem driven? The VALIDATE approach states that health technologies are a sort of policy solutions proposed to address particular health problems, and HTH should always critically assess the underlying problem definition (1; 7; 28; 29) |

# Closure

Finally, do you have any questions for me? Is there anything else that you would like to comment on that I haven’t already asked you about?

[Stop audio recording]

We will write a summary of the interview. Could we ask you to provide feedback on this summary, and check whether we have interpreted your answers correctly, or add additional information to answer our questions?

The results of the analysis of the interviews will be submitted for publication in a peer-reviewed journal, and you will be informed about this. We may ask you to clarify certain answers and / or provide comments to our analysis.

Another part of our study is a survey on procedures and methodology for medical devices in general. Did you see the invitation? Would you, or colleagues, be willing to fill out the survey?

Do you know a colleague that we should invite for an interview to gain more information about HTA of medical devices?

If you have any remaining questions or comments, feel free to contact us.

Thank you for your participation and help!

# References

1. van der Wilt G.J., Bloemen B., Grin J., et al. Integrating Empirical Analysis and Normative Inquiry in Health Technology Assessment: The Values in Doing Assessments of Health Technologies Approach. International Journal of Technology Assessment in Health Care, 2022, 38(1).

2. Van Oudheusden M., Delvenne P. & Lucivero F. Making the invisible visible. TATuP Zeitschrift für Technikfolgenabschätzung in Theorie und Praxis, 2019, 28(1), 21-26.

3. Torgersen H. Three myths of neutrality in TA - How different forms of TA imply different understandings of neutrality. Technological Forecasting and Social Change, 2019, 139, 57-63.

4. Gagnon H., Legault G.A., Bellemare C.A., et al. How does HTA addresses current social expectations? An international survey. Int J Technol Assess Health Care, 2020, 37, e9.

5. Culyer A. & Husereau D. Redefining Health Technology Assessment: A Comment on "The New Definition of Health Technology Assessment: A Milestone in International Collaboration". Int J Technol Assess Health Care, 2022, 38(1), e54.

6. O'Rourke B., Oortwijn W. & Schuller T. Response to redefining health technology assessment: a comment on "the new definition of health technology assessment: a milestone in international collaboration". Int J Technol Assess Health Care, 2022, 38(1), e55.

7. Lehoux P. The problem of health technology. New York: Routledge, 2006.

8. Goetghebeur M.M., Wagner M., Samaha D., et al. Exploring Values of Health Technology Assessment Agencies Using Reflective Multicriteria and Rare Disease Case. Int J Technol Assess Health Care, 2017, 33(4), 504-20.

9. Bauer A. & Kastenhofer K. Policy advice in technology assessment: Shifting roles, principles and boundaries. Technological Forecasting and Social Change, 2019, 139, 32-41.

10. Ducey A., Ross S., Pott T. & Thompson C. The moral economy of health technology assessment: an empirical qualitative study. Evidence & Policy: A Journal of Research, Debate and Practice, 2017, 13(1), 7-28.

11. Harvard S. & Werker G.R. Health Economists on Involving Patients in Modeling: Potential Benefits, Harms, and Variables of Interest. Pharmacoeconomics, 2021, 39(7), 823-33.

12. Brazier J.E., Rowen D., Lloyd A. & Karimi M. Future Directions in Valuing Benefits for Estimating QALYs: Is Time Up for the EQ-5D? Value Health, 2019, 22(1), 62-68.

13. Enzing J.J., Knies S., Boer B. & Brouwer W.B.F. Broadening the application of health technology assessment in the Netherlands: a worthwhile destination but not an easy ride? Health Econ Policy Law, 2020, 1-17.

14. Fuchs S., Olberg B., Panteli D., Perleth M. & Busse R. HTA of medical devices: Challenges and ideas for the future from a European perspective. Health Policy, 2017, 121(3), 215-29.

15. Enzing J.J., Vijgen S., Knies S., Boer B. & Brouwer W.B.F. Do economic evaluations of TAVI deal with learning effects, innovation, and context dependency? A review. Health Policy and Technology, 2021, 10(1), 111-19.

16. Pomey M.P., Brouillard P., Ganache I., et al. Co-construction of health technology assessment recommendations with patients: An example with cardiac defibrillator replacement. Health Expect, 2020, 23(1), 182-92.

17. Steffensen M.B., Matzen C.L. & Wadmann S. Patient participation in priority setting: Co-existing participant roles. Soc Sci Med, 2022, 294, 114713.

18. Staniszewska S. & Soderholm Werko S. Mind the evidence gap: the use of patient-based evidence to create "complete HTA" in the twenty-first century. Int J Technol Assess Health Care, 2021, 37, e46.

19. Bidonde J., Vanstone M., Schwartz L. & Abelson J. An institutional ethnographic analysis of public and patient engagement activities at a national health technology assessment agency. Int J Technol Assess Health Care, 2021, 37, e37.

20. Cleemput I., Dauvrin M., Kohn L., et al. Developing an agency's position with respect to patient involvement in health technology assessment: the importance of the organizational culture. Int J Technol Assess Health Care, 2020, 36(6), 569-78.

21. Moes F., Houwaart E., Delnoij D. & Horstman K. Contested evidence: a Dutch reimbursement decision taken to court. Health Econ Policy Law, 2017, 12(3), 325-44.

22. Wale J.L., Chandler D., Collyar D., et al. Can We Afford to Exclude Patients Throughout Health Technology Assessment? Front Med Technol, 2021, 3, 796344.

23. Moes F., Houwaart E., Delnoij D. & Horstman K. Questions regarding 'epistemic injustice' in knowledge-intensive policymaking: Two examples from Dutch health insurance policy. Soc Sci Med, 2020, 245, 112674.

24. Hulstaert F., Pouppez C., Primus-de Jong C., Harkin K. & Neyt M. Evidence gaps for drugs and medical devices at market entry in Europe and potential solutions. KCE Reports. Brussels: Belgian Health Care Knowledge Centre (KCE); 2021.

25. Ming J., He Y., Yang Y., et al. Health technology assessment of medical devices: current landscape, challenges, and a way forward. Cost Eff Resour Alloc, 2022, 20(1), 54.

26. Tarricone R., Torbica A. & Drummond M. Challenges in the Assessment of Medical Devices: The MedtecHTA Project. Health Econ, 2017, 26 Suppl 1, 5-12.

27. Pongiglione B., Torbica A., Blommestein H., et al. Do existing real-world data sources generate suitable evidence for the HTA of medical devices in Europe? Mapping and critical appraisal. Int J Technol Assess Health Care, 2021, 37(1), e62.

28. Kluytmans A., Tummers M., van der Wilt G.J. & Grutters J. Early Assessment of Proof-of-Problem to Guide Health Innovation. Value Health, 2019, 22(5), 601-06.

29. Giacomini M., Winsor S. & Abelson J. Ethics in Health Technology Assessment: Understanding Health Technologies as Policies. Healthcare Management Forum, 2013, 26(2), 72-76.
