# Supplementary material for: Assessing medical devices: a qualitative study from the validate perspective
Source: Int J Technol Assess Health Care. 2024 Apr 24;40(1):e29. doi: 10.1017/S0266462324000254 (PMC11569912; doi:10.1017/S0266462324000254)
Supplement: Bloemen and Oortwijn supplementary material 4 — Bloemen and Oortwijn supplementary material [file S0266462324000254sup004.docx]

Supplementary file 4 – Supplementary Figures and Tables


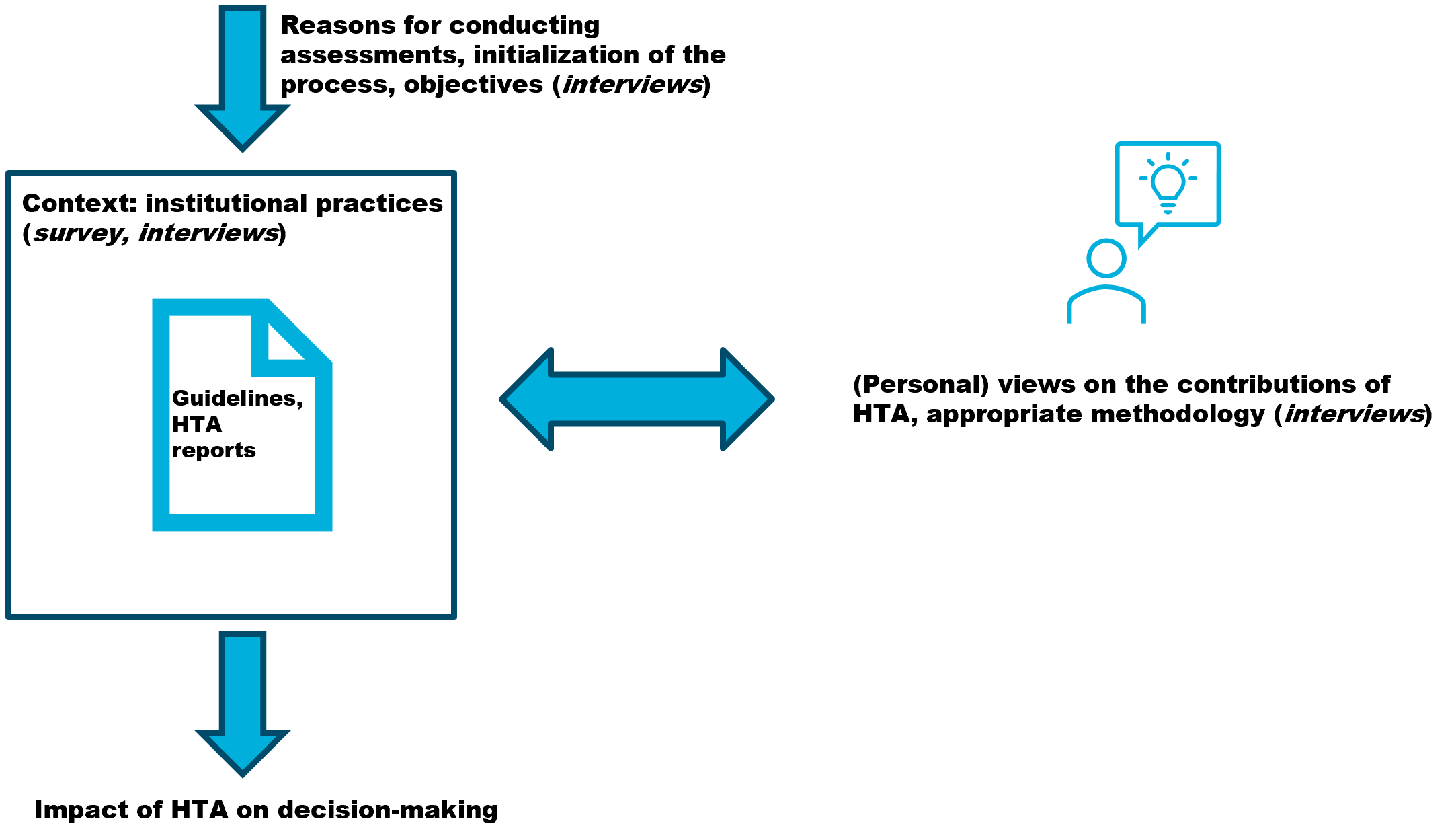


**Supplementary Figure 1. Schematic illustration of the qualitative approach taken in this study**. This qualitative study used surveys to obtain information about procedures used at HTA agencies for initializing and conducting assessments (‘institutional practices’) and the methodology guidelines used for conducting assessments of (high-risk) medical devices. Interviews were conducted to obtain more in-depth information for reasons behind the prioritization and initialization of assessments, the procedures, methodology; and to obtain information about the personal views of HTA practitioners on the role of HTA, its contribution to society, and appropriate methodology for realizing that.

**Supplementary Table 1.**Overview of interviews conducted and characteristics of the agencies where interviewees are working (institutional context) and their process (initialization, scoping) for assessing medical devices.

| **Institution, country / region** | **Focus of interview; date** | **How are devices selected and identified for HTA?** | **How is the scope of an assessment determined?** | **Are the results of scoping reported?** |
| --- | --- | --- | --- | --- |
| **Ontario Health, Canada** | HTA report on TAVI; 13-02-2023 | Topics for HTA review can be suggested by any person or organization from across Ontario through an open application process. Ontario Health receives many topic suggestions for assessments, and there is a prioritization process to select which requests are addressed. | The scope centers upon the request submitted in the HTA application, and is further developed by peer-reviewing published literature and grey literature, consulting with stakeholders (e.g. patients, clinical experts, and representatives from the provincial government, the health system, and industry). The PICO(TS) tool is used to help develop the research questions for the HTA. The scope may include equity issues, which are identified with the PROGRESS-Plus framework. | No. The scoping phase is not described in the HTA report, there is a *clinical review plan*, which is a written record of the context, rationale, and plan for the clinical evidence review, but this is an internal document. |
| **HIQA, Ireland** | HTA report on TAVI; 23-02-2023 | HTAs are typically formally requested by the Minister for Health or the HSE; topics may also be suggested by service providers or users, anyone can contact HIQA directly to suggest a potential HTA topic. A prioritization process is used to select HTA topics for which an assessment will be conducted. Topics are ranked based on high-level information on four areas of impact: clinical, economic, policy and organizational. An HTA Prioritisation Advisory Group, comprising of key stakeholders (Department of Health, HSE) is involved in selecting the most relevant topics, with being linked to an imminent decision being the main criterion. | Initial review to determine the type and extent of information available on the topic, and to check whether HTAs have already been conducted on the same topic in other countries. In collaboration with the decision-maker, it is decided which questions will be answered in the assessment; depending on the information that is needed to support a decision, the scope can be narrow or broad. The resulting terms of reference for the assessment are reviewed by an Expert Advisory Group, consisting of representatives from relevant stakeholders. | The members of the Expert Advisory Group and the terms of reference for the assessment are reported in the final HTA report. |
| **ZIN, The Netherlands** | Survey on HTA of medical devices; 06-04-2023 | Formal request by decision-makers, horizon scanning; no formal criteria, assessment can take place in case there is a legal or nation-wide societal dispute, initiated by the agency. | The scope is already defined by law, i.e. medical devices are only assessed when there are doubts about fulfillment of the legal requirement of health services to be part of ‘established medical science and clinical practice’, which implies an assessment of its clinical effectiveness; they can also be indirectly assessed when they are part of an intervention that makes use of the device, and the intervention needs assessment, to which respective legal frameworks and guidelines apply. | No scoping process. |
|  |  |  |  |  |
| **Avalia-t / ACIS, Spain, Galician region** | Survey on HTA of medical devices; 12-04-2023 | Formal request by decision makers, open consultation of different stakeholders, horizon scanning; use of Pritec tool (developed by Avalia-t) that prioritizes devices for assessment based on clinical, economic, ethical, social and legal criteria. | The PICO(D) tool is used to define the objectives of the assessment, stakeholders can provide feedback. | The PICO(D) and objectives of the HTA are mentioned in the final HTA report. |
| **CDE / HTA, Taiwan** | Survey on HTA of medical devices; 14-04-2023 | Formal request by decision makers; if the budget impact likely exceeds 30 million New Taiwan Dollars (NTD), the CDE needs to assess the device. | No public guidelines. | HTA reports are only published in Mandarin Chinese. |
| **IETS, Colombia** | Survey on HTA of medical devices; 17-05-2023 | Formal request by decision makers (Ministry of Health). An assessment is only initiated when there is an expectation that it will have a significant budget impact. | PICO an TICO tool; literature review; focus groups and interviews with stakeholders. Safety and clinical effectiveness are always assessed, health economic analysis (budget impact) is only performed when it is specifically requested by the decision makers. Colombian law states that effectiveness, safety and quality of the evidence need to be assessed. | HTA reports are only published in Spanish. |

**Notes:** Avalia-t / ACIS: Unidad de Asesoramiento Científico-técnico (Avalia-t); CDE/HTA: Center for Drug Evaluation Health Technology Assessment; HIQA: Health Information and Quality Authority; IETS: Instituto de Evaluación Tecnológica en Salud; ZIN: Zorginstituut Nederland.

**Supplementary Table 2.** Overview of answers provided to survey questions on initialization of assessments of medical devices.

| Question | Answers | Percentage |
| --- | --- | --- |
| *Please can you specify what definition of medical devices (e.g. in line with current EU-directives) your (national/regional) HTA agency uses?* [open question] (n=15) | *EU MDR (n=4)*  *Often the definition of EU-directives is considered. There is however a distinction made between in-hospital medical devices (intramural) and outpatient medical aids (extramural).*  *We do not use a specific definition of medical devices. They are understood as an instrument, tool, machine, test, implement, or implant used to prevent, diagnose or treat disease or other conditions.*  *We conduct HTA on 'medical devices and clinical interventions' so we will conduct HTA on anything 'non-drug' used in patient care following the definition of a health technology.*  *Pharma Single Technology Assessments are done by another national agency, while we are responsible for Single Technology Assessments on medical devices and multitechnology assessments, including pharma and medical devices.*  *Anything that is not a medicine/pharmaceutical. Included, non-med technologies, devices, diagnostics, procedures, models of care etc. we also assess social care.*  *Any invasive or non-invasive medical products and appliances used to improve health services and health outcomes.*  *Devices used by medical personnel to treat patients in clinical settings.*  *In our country, devices are not assessed as individual products, but only in the context of their use within medical procedures related to a specific indication; only devices that have a CE mark are eligible, therefore, the definition does not differ; however, decision-making does not relate to specific products, but to procedures and a generic description of the device used with it.*  *If the medical devices budget impact analysis increases more than US $1 million, new functional categories need to be reviewed by us. And only for special materials, included implantable (e.g., stent, artificial hip joints)and specific non-implantable (e.g., aortic balloon catheter).*  *In-line with our National Device Act from 2012.*  *We have our own definition that follows our national law.* | |
| *Does the process to assess medical devices differ from the assessment of pharmaceuticals at your (national/regional) HTA agency?* (n=15) | Yes | 67% |
|  | No | 33% |
| *What are the differences between assessments of medical devices and pharmaceuticals?* (n=10) | *Criteria to evaluate, some points to derive from data, differences in efficacy and effectiveness measuring.*  *Not all medical devices have to be assessed as an HTA, while all pharma have to be evaluated to be introduced in our healthcare system. Whereas pharma are assessed as Single Technology Assessments (STAs) based on documentation submitted by Industry, medical devices can be STAs, but are usually as multiple technologies assessments. In that last case, documentation is gathered through literature searches.*  *Our agency is focusing on medical device only.*  *Device and drug HTA are conducted using the same general principals, but some components are incorporated regularly into device HTA that may not be included in drug HTA - such as implementation considerations, environmental considerations, legal issues.*  *Pharmaceutical assessment focuses on the additional benefit for the purpose of price negotiations, but drugs are available on the market anyway, while the assessment of high-risk device-related procedures aim to determine whether evidence is sufficient in order to accept those technologies in health care.*  *Medical devices are assessed by RedETS and medicinal products are assessed by AEMPS and Revalmed (another Spanish network).*  *Differs on sources of information and synthesis (not Systematic review), time to final report (3 months), more involvement of professionals and patients).*  *Both are performed by the same agency, but they have two different committees for the evaluations. The requirements are different between the two. For medications the agency requires evidence from clinical trials. For devices they only require technical information and information that support the safety of the device. However, this last information does not need to be from clinical trials and can be only analytical.*  *Yes, but assessment of pharmaceuticals is done by another agency in our country. Key difference is that assessment of pharma is based on manufacturer submissions and non-pharma on rapid reviews typically with de novo economics.*  *Explicit assessments usually involve only effectiveness assessment and take place only for a limited number of devices.* | |
| *What are mechanisms for identifying medical devices for HTA in your country / region?* [multiple answers possible] (n=15) | Formal request of decision-makers (e.g. Ministry of Health, hospital boards) to HTA agency | 73% |
|  | Industry submission to HTA agency | 47% |
|  | Horizon scanning systems | 47% |
|  | Other, please specify:  - Members of consortia (n=1)  - Online platform where anyone can submit a topic; commissioning board decides (n=1)  - New HTA: industry submission; re-assessment: mixed (mainly formal request from decision makers, selection based on internal screening, open consultation of clinical professionals) (n=1)  - submission by hospitals (n=1)  - All of the other mechanisms and themed topic calls and stakeholder engagement (n=1) | 33% |
|  | Consultation of different stakeholders regarding pre-selected areas | 20% |
|  | Open consultation of different stakeholders | 20% |
|  | At the remit of the HTA agency | 20% |
| *What criteria are used for the selection of medical devices for assessment?* [multiple answers possible] (n=15) | Economic impact / costs | 60% |
|  | Potential health benefits | 60% |
|  | Importance to health care | 53% |
|  | Other, please specify:  - N/A (n=1)  - no formal list of criteria, decided by the board on a case by case basis (n=1)  - safety, effectiveness, feasibility of (re-)assessment (n=1)  - Not yet reimbursed (n=1)  - Prioritization tool that is developed by HTA agency (n=1) | 53% |
|  | Burden of disease | 47% |
|  | Population size | 40% |
|  | Ethical aspects | 27% |
|  | New indication | 27% |
|  | Innovativeness | 27% |
|  | Social, cultural and/or legal aspects | 20% |
|  | Indication extension | 13% |

**Supplementary Table 3.** Consolidated results of the thematic analysis of the interviews: identified subthemes, the total number of associated fragments for the themes and subthemes, and illustrative fragments from summaries of the interviews.

| Themes | Subthemes | Fragments from summary of interview |
| --- | --- | --- |
| Scoping (13 fragments) | Objectives, methods and processes of scoping (7 fragments)  Scoping process itself is not reported (2 fragments)  The problem definition underling current clinical practice is not questioned or difficult to evaluate (4 fragments) | *Our recently updated HTA methods manual includes information on scoping, but for every assessment the scope needs to be specified. Scoping of the topic is performed before the start of every assessment. In the scoping phase, we try to understand the topic by looking at the literature and talking with stakeholders (e.g. Ministry, stakeholders involved in the implementation, clinicians, manufacturers, other stakeholders), and try to determine the PICO (Population, Intervention, Comparator, Outcome) question. For transparency, all manufacturers that produce a device similar to the topic of the assessment are involved to give them the opportunity to provide their perspective. The goal is to understand the perspectives of the different stakeholders, and to identify any issues with equity, ethics, feasibility (implementation issues). […] During the scoping phase we try to identify any issues associated with the use of a technology, including societal and feasibility issues. All the issues identified in literature or in conversations with stakeholders are mentioned in the HTA report. This includes looking at preferences of healthcare providers or patients.* [Interview #3]  *Normally, requests for topics come from the ministry or healthcare management. We also encourage clinicians to send in requests. Currently, the demand for assessments exceeds our capacity. Therefore, based on all received requests, topics are prioritized. An important criterion in this is that a decision-maker has a need for a particular assessment. […] According to our guidelines, we carry out an initial review to determine the type and extent of information available on the topic, and check whether HTAs have already been caried out on the same topic in other countries. Working with the decision-maker, we determine what questions will be answered in the assessment. A full HTA collects and summarizes evidence under a number of fields or domains, which include: description of the technology, burden of disease, clinical effectiveness and safety, cost-effectiveness and budget impact, organizational and social aspects, ethical and legal issues. Depending on the information that is needed to support a decision, the scope can be narrow or broad. […] As part of the prioritization process, we often provide an initial recommendation about what is required for the topic. For some topics, we will conclude that there is insufficient evidence to support an HTA or that the only information needed is on clinical effectiveness. If it is agreed upon that an HTA is needed and possible, it is discussed with the decision-maker what information is needed for them to make a decision. The outcome of this is the terms of reference for the report, and stakeholders are asked to provide input (e.g. do they miss anything?).* [Interview #6]  *The scoping phase is not described in the HTA report, there is a clinical review plan, which is a written record of the context, rationale, and plan for the clinical evidence review, but this is an internal document.* [Interview #3]  *The terms of reference for the assessment of TAVI are stated in the report. These terms of reference were reviewed by the Expert Advisory Group, a group of representatives from relevant stakeholders that is formed to support an assessment.* [Interview #6]  *Not for TAVI for low surgical risk patients, because at the time of the HTA SAVR was considered to be the proper comparator as it was considered the standard of care according to experts in the field. If there would be another relevant comparator, that intervention would already have been tried in the treatment of these patients. And at the time of the HTA, patients at this stage of the disease always received SAVR. We don’t question this golden standard in clinical practice. […] Not in the case of TAVI because no other relevant comparator was identified during scoping and this was validated by experts in the field. Additionally, the quantitative and qualitative preferences literature, and engagement with patients, did not identify any other relevant comparators.*  [Interview #3]  *Yes, we are addressing general problems already. Example: therapeutic positioning reports in Spain. We are doing, for almost 10 years, ‘therapeutical ranking’. For example, ranking all interventions for asthma, and making recommendations, based on the type of asthma, which intervention is best. But the problem with these types of analyses is that this demands a high number of professionals (including clinicians) working on it, requiring a lot of time and resources. The future of these type of analyses would depend on cross-country collaborations in HTA. […] AI could be helpful in this, reducing the time needed for systematic literature searches, although some standardization of methodology would be needed to implement that.* [Interview #5]  *Yes, it might become more system wide. For example, can you look at the care for diabetes in our country? But these type of questions tend to become unanswerable, because the evidence base is so broad that you have no means to summarize it in a timely manner. Because of the requirements of scientific rigor, you can not look at the whole system. In addition, we have limited capacity to do this.* [Interview #6] |
| The use of different types of evidence in assessments of medical devices (23 fragments) | The evidence requirements for medical devices are not different from drugs (9 fragments)  The importance of comparative data (3 fragments)  Real-world data / evidence could be useful to address particular issues of devices (5 fragments)  Qualitative research (6 fragments) | *Only RCT data was used to assess TAVI, the real world data is not used to make decisions on reimbursement, and we use registry data and real-world data to monitor after reimbursement.* [Interview #1]  *No, it’s not a black and white matter. There is some recognition at HTA agencies that real-world data and observational data should be considered in assessments. How I see it is that it renders a methodological inquiry rather than a concern on neutrality and impartiality. The challenge is in integrating these approaches in assessments while simultaneously adhering to the current legal frameworks which are still focused on RCT data. But which types of data are used should depend on the type of questions raised by an assessment.* [Interview #2]  *The requirements on evidence for assessing medical devices should not be different from those for assessing drugs. However, for medical devices the availability of RCTs is often limited, but we always use the highest level of evidence that is available for a given outcome. Therefore, observational data and real-world data can be used to assess medical devices when deemed appropriate*. […] *The use of observational and / or real-world data for assessing TAVI was part of the discussion before the methodology and literature search was finalized (it was determined during the scoping phase). If observational studies provide information on the same outcomes and for the same follow-up duration as RCTs, and RCTs are of high quality (no risk of bias), RCTs are preferred because they are higher in the hierarchy of evidence. If RCTS are available, observational studies are considered only if they provide additional information to RCTs (i.e. in terms of types and/or duration of outcomes, e.g. longer-term outcomes) or if observational studies are of comparable quality to RCTs. In the case of TAVI, there were two high-quality RCTs available and no information was missed, i.e. there were no observational studies known that could add any relevant information.* [Interview #3]  *The future of RCTs is not that big, for many things it is not useful or too expensive to perform. Data collected under different circumstances, using correct analyses, can be really important as an alternative. We have used cohort data and causal methods using marginal analysis to do assessments. What is important is to have a valid method, irrespective of the source of the data.* [Interview #5]  *“We start at the top and work our way down”. Our approach is took at the best available evidence, and sometimes there is only low quality evidence available. The poorer the quality of evidence, the higher the uncertainty, but we don’t exclude it. If case studies are the only type of evidence that is available, we will use it, but that means high uncertainty. […] Yes, if it is an important aspect of the decision, even though the evidence is poor, we will address it. But is important to be honest about the evidence that is available and the conclusions you can draw. So, if needed, mention that there is no inconclusive evidence available. […] We don’t make different choices. Whether it is a drug, device or public health intervention, we still need to decide what evidence the decision-maker needs, and we use the same evidence hierarchy – we still make use of the best available evidence.* [Interview #6]  *There is some confusion about the definition of ‘real-world evidence’. People talk about different things when using this term. Important is that we use comparative data, i.e. we need information on how the effects of a technology compares to other possibilities (e.g. conventional treatment) because it is otherwise difficult to interpret its meaning. What is also important is to consider the risk of bias in data. Real-world data can be important to understand the effects of particular devices in clinical practice, but we need to take comparative data and risk of bias into account.* [Interview #3]  *What we try to do to address these challenges with medical devices is to make comparisons (e.g. comparing outcomes of interventions using different devices), because that is really important. […] Because, from the perspective of the decision-maker (Ministry of Health) you are focused on the health of the population and the healthcare system, not on a single device. You need information that allows you to compare different technologies to make decisions on that level, to know what you sacrifice if you decide to invest in a particular technology (because resources are limited).* [Interview #5]  *Yes, real-world evidence should be used in the assessment of (especially high-risk) medical devices. A problem is that devices are continuously changing, and data is often from first generation devices. Therefore, it is important to use real-world evidence.* [Interview #1]  *The standard outcome measures, and associated research designs and analytical tools, were created for assessing pharmaceuticals, whereas this methodology is not always feasible for medical devices. One major challenge with devices is that they don’t work for themselves, its effectiveness depends on the appropriate ecosystem (e.g. existence of other, supporting, devices and services) and the user (e.g. differences between surgeons in how they perform a particular procedure). Another challenge is that devices could potentially have a long-term use or effects, but studies only have a short time horizon.* […] *Yes, we are going to use these methods for all types of interventions. For example, for personalized medicine it is not possible to perform RCTs. The challenge for the future is how to combine all these different types of information.* [Interview #5]  *“Real-world data is a red herring, it is just data”. Putting the label ‘real-world data’ makes it sound as if it is something better than observational data, whereas it is similar. And we need to be aware of its biases, and not trying to sell it as if it is more than it is. But observational data is important. There are particular issues with respect to medical devices: the data collected in RCTs is often of a small sample size and is not applicable to the current situation due to iterative development of devices. This means that the landscape is currently changing, i.e. when you conduct a clinical effectiveness analysis, the data that you collect (in a systematic review) are based on devices that bear almost no resemblance with the devices that are available now. The evidence base does not match the device that your are actually assessing. That is a major problem, and there is no clear solution. At least it requires an understanding of the details of the device and what are the refinements of the device over time. For this information we are dependent on the knowledge of the clinicians and other experts involved in developing the device, who may be biased.* [Interview #6]  *Not in assessing clinical effectiveness, but in determining the scope of HTA, in eliciting stakeholders’ perspectives, especially patients, and in understanding the context of health technology the use of qualitative data could be quite informative.* [Interview #2]  *Yes, it is important and when deemed necessary a quantitative and/or qualitative patient and/or healthcare professionals preference review are done. But qualitative research needs a specific training, which is not my expertise. Therefore, difficult to evaluate its validity, and there is a need to ask external experts. For quantitative research its validity is easier to understand (e.g. GRADE tool for evaluating validity). But quantitative and qualitative research is both important. And for both it is important to understand risk of bias and its validity.* [Interview #3]  *Yes, for assessing patient / client satisfaction. For example, it could be that for laparoscopy there are two devices with a similar price and effectiveness, but the user experience (e.g. surgeon) is different, and it can be important to take that into account.* [Interview #5] |
| Aspects considered in assessments of medical devices (16 fragments) | Only safety and clinical effectiveness are always considered (5 fragments)  Challenges with considering quality of life, ethical aspects (11 fragments) | *Quality of life depends on the medical device. We can’t have the quality of life evidence for every medical device. In general, the outcomes depend on the device. […] We look at RCTs, and if not available we use observational studies. If they have reported on quality of life we will include the information in the report, but we do not only focus on it. […] I do think that patient experiences and quality of life is important as a reference for reimbursement decisions, but we do not just focus on patient opinions during the assessment and do not use quality of life as a search key word.* [Interview #1]  *We try to draw neutral conclusions on safety and efficacy. And we try to identify whether there are any evidence gaps (e.g. non-reported outcomes), and whether it is needed to redo the assessment (i.e. because there are ongoing trials). […] We have no experience with health economics modeling, because these are not conducted by ourselves. When a health economics model is needed, other HTA agencies in our country are contacted that have wide experience and staff for health economics models are asked for help. We have hired an economist recently but he / she has no experience in economic assessments of health issues and is currently receiving training on that.* [Interview #4]  *The scope of an assessment is primarily determined by our national law that states that effectiveness, safety and quality of the evidence need to be assessed. A budget impact analysis can also be conducted.* [Interview #5]  *Although the relevance of ethical analysis is acknowledged, in practice it is mostly not conducted. Important barrier is that the assumption is that it is sufficient that clinicians, health economists, epidemiologists, HTA practitioners, can take ethical aspects into account as part of their analysis. So it is not recognized as a separate domain or analysis step. There is no strong perceived need for an ethicist being explicitly involved in these domains, or a formal integration of an additional ethical analysis. […] It seems to be no one’s concrete responsibility, or all stakeholders (HTA practitioners, decision-makers etc.) refer to each other. There are different views about what is the appropriate place to address this, some would say that it is the responsibility for political parties or decision-makers.* [Interview #2]  *“it is our usual process, especially in more recent years, to assess whether an ethics review is warranted using a trigger tool – and this is probably why I mentioned it. I don’t think that this was done at that point for TAVI”. […] During the scoping phase, when talking with clinicians and stakeholders, we validate whether we have identified all the relevant outcomes. Autonomy and well-being are important outcomes and may be included as outcomes for instance as part of quality of life, but no experience with autonomy as a separate outcome in an assessment. In the case of TAVI, in the quantitative preferences review, something about the influence on daily life came up. So, that is important, but difficult to include it explicitly as an outcome. It can also be assessed in the patient preferences and values review.* [Interview #3]  *Our priority is to use systematic reviews in order to produce the HTA report in a timely manner, this includes reviewing safety and effectiveness studies. For assessing the preferences and views of patients, qualitative studies are used. Or patient groups and societies are contacted. The evidence collected is summarized with GRADE. In case of qualitative studies, the CASP and GRADE-Cerqual are used. In case of technologies with high ethical impact, we can request ethical experts as established in their methodological guidelines. […] It could be a good idea to include other outcome measures. In general, the quality of studies on the effects of medical devices, including the patient perspective, is improving. It could be important to include this, because the involvement of the patient can influence the safety, efficacy and costs of the device.* [Interview #4]  *We do highlight issues when there are serious inequity implications of adopting particular interventions. But we are not in the position to change anything about the private part of the healthcare system. […] Sometimes decisions are based on things like political expediency, or some other reasons that we cannot capture as part of the evidence base. For example, in the case of orphan drugs, which are not cost-effective, there may be reasons to reimburse them because of care for a group of people who don’t have other options. But an HTA struggles to capture that information because it is very hard to do that objectively, although we can highlight it under patient, social and ethical issues. It is not the role of an HTA agency to get everything that is required for the decision, we have to look at the things we can manage objectively.* [Interview #6] |
| Stakeholder involvement in assessments of medical devices (24 fragments) | Stakeholder involvement poses challenges to the objectivity of HTA (7 fragments)  Stakeholder involvement is challenging due to their limited understanding of the HTA process (3 fragments)  Potential contributions of stakeholder involvement (10 fragments)  Stakeholder contributions have limited or unclear impact on recommendations and decisions (4 fragments) | *The people doing the HTA need to be objective, analyzing the evidence. Any person with conflicts of interest, or any particular relation to the topic of the report (e.g. family members with the disease), can have biases. But we have to produce the HTA reports in an objective way (as most as possible), without any influence of manufacturers or clinicians that are influenced (funded) by manufacturers. The members of the committee that makes the decision, especially in these cases, should be independent from influences by manufacturers. Although it is unavoidable that HTA practitioners put something of their personal perspective in the report, they should be free of conflict of interest and try to be as objective as possible. […] The problem is that manufacturers try to influence assessments via patient associations and patient input. It is sometimes difficult to know whether patient organizations have these attachments with manufacturers leading to conflicts of interest. But we are also dependent on manufacturers, patients and clinicians to obtain certain information. Therefore, we try to review, when receiving input or comments from manufacturers, whether there are any biases. We carefully look at which comments or input improves the quality of the HTA report, this means assessing benefits of the medical device from a societal viewpoint and excluding benefits to particular perspectives. […] Patients and clinicians can provide many information during the protocol phase. They provide feedback on revisions of the HTA reports, or participate in the group that elaborates the HTA report. Criteria for inclusion is that they don’t have any conflicts of interest (their level of involvement depends on this).* [Interview #4]  *The interviewee recalls an experience with a patient telling about her experience with a particular intervention. She explained how burdensome it was for her. According to the interviewee, there is no literature that would provide the same information. But there are no means to turn that experience into a number so that we can say ‘the answer is do this’. This is always very challenging. The HTA practitioner needs to be as honest as possible about the information available. If information is received from patients a way to incorporate has to be found. But a single story of a patient is similar to a case study, which can be misleading. The risk is of being overwhelmed with information. [..] Therefore, asking patients whether they can recall a particular experience (prompted by anectodical evidence) may lead to confirmation bias. We can not base conclusions on anectodical evidence. What we can do is saying that there is some evidence that some patients are unhappy with the intervention, but that it is unclear whether that is a general experience. […] But we have to be careful that we don’t end up with people that are gaming the system, it is important that the evidence is impartial. And it is important that people think about the greater good. For example, in the case of TAVI, you could invite a patient representative of people with heart complaints. But should you not also include (representatives of) all other patients (besides aortic stenosis patients) that are affected because resources are used for TAVI? Shouldn’t they be able to say that we actually get very litter for our money, what about the rest of us? So you need to be careful that you get a balance.* [Interview #6]  *We have been engaging the community and stakeholders in our analysis, but this is hard because people in our country are not used to being involved in these analyses. Therefore, we have been training patients and families about HTA. In addition, the results of an HTA are presented to panels consisting of healthcare professionals that are going to use the device, stakeholders (excluding industry), and the government. These can provide feedback on the results. And a bioethicist and lawyer are usually part of an HTA team, conducting an ethical analysis within the limits of our national law.* [Interview #5]  *In the case of pharmaceuticals, manufacturers are very clever and know how to involve patients to maximize the chances of a good outcome. For medical devices the manufacturers are not that mature yet, and they involve patients to tell them what is important to them. Only patients can tell you what is important them, and patients are the ones you ultimately want to help. But this needs education, to inform patients about how HTA processes works, and which evidence is required. But it can only be for the good of HTA if patients are more involved and have a better understanding of what is required.* [Interview #6]  *Sometimes, in HTA reports in other countries patients are consulted as experts, and that information will be used. And patients’ opinions are collected for at least 30 days before the application is listed on the agenda for the committee meeting. […] All items for assessment are posted on an online platform. On this platform, patients can provide input to the assessment, in terms of their preferences and experiences. Thirty days before a committee meeting is scheduled all the provided input is collected and summarized by our HTA division, and findings sent to the committee for consideration. Patients, caregivers and patient organizations can share seven kinds of information: (i) the method of information gathering (how do you gather opinions?); (ii) experiences of living with the conditions / diseases; (iii - iv) experiences of the traditional and new treatments; (v) expectations regarding the new treatments; (vi) effects on caregivers with / without the new treatments; (vii) other opinions. In addition, guidelines were developed to help patients get their voices heard, and we were involved in educational programs for patients, caregivers, volunteers in hospitals, and patient organizations to educate them about HTA, the reimbursement process, and the patient involvement mechanisms.* [Interview #1]  *In our country, the HTA report is used for reimbursement decisions. When conducting an assessment, we think about the benefits of a technology for society. This means it is important that there is a link with potential benefits for the patients. […] The patient is the most important stakeholder, but not the only one. The perspective and satisfaction of the clinician is also important. For a good use of medical devices, the clinicians and patients are both needed. Both influence the safety and efficacy of medical devices. […] We have to focus on the issues considered relevant by Ministry of Health, both specific issues as a given medical device or wider as pseudo therapies assessments directed to avoid population use them instead of their treatments.* [Interview #4]  *What is important is that experts in HTA methodology engage more with stakeholders, to explain them wat we are doing. If people do not know what we are doing, and why, it is difficult to address the problem of low value technologies that are still being introduced or used.* [Interview #5]  *Given that HTA professionals are not topic experts, but methodological experts, we need to inform the clinical and patient population about the kinds of information required to be able to draw conclusions. In every assessment, there is involved an Expert Advisory Group with members from decision-making, service providers, clinicians or other professions that are impacted, patient representatives. Sometimes members say that they have a different experience. Our response is always “show us the data” that supports your point of view. And sometimes they provide local data to show their point of view. But often they act upon a hunch and cannot provide any data. […] But observational data is important. There are particular issues with respect to medical devices: the data collected in RCTs is often of a small sample size and is not applicable to the current situation due to iterative development of devices. This means that the landscape is currently changing, i.e. when you conduct a clinical effectiveness analysis, the data that you collect (in a systematic review) are based on devices that bear almost no resemblance with the devices that are available now. The evidence base does not match the device that your are actually assessing. That is a major problem, and there is no clear solution. At least it requires an understanding of the details of the device and what are the refinements of the device over time. For this information we are dependent on the knowledge of the clinicians and other experts involved in developing the device, who may be biased.* [Interview #6]  *Actually, the patients have the feeling that their opinions do not really influence the decision, that their opinion is not valued in reimbursement decisions. It should be discussed during the committee meeting, but we cannot control it. In the meeting, the opinions are most often only collected from clinical experts, and there is a discussion on budget impact and clinical benefit. The focus is primarily on discussing budget impact, that determines the decision. Because the costs of healthcare in our country are on the rise, it is very difficult to ignore budget impact. During the committee meeting a patient is invited. But most often there are clinical experts discussing the clinical benefit and budget impact, which are seen as the most important aspects.* [Interview #1]  *Information from RCTs is not the only factor considered in the deliberations process of making a recommendation. Factors considered include effectiveness, safety, patient preferences and values, equity, cost-effectiveness, budget impact, and feasibility. Additionally, feedback received on the draft recommendation from different audiences (patients, health care professionals, organizations, general public, etc.) is also considered when making the final recommendation. I am not involved in making such decisions, but I understand that the interplay of these factors is specific to each decision / technology, so it is not possible to have a statement about what should be done in general.* [Interview #3] |

**Supplementary Table 4.** Overview of answers provided to survey questions on aspects considered in assessments of high-risk medical devices.

| Question | Answers | Percentage |
| --- | --- | --- |
| *Which aspects are considered by your HTA agency when assessing high-risk medical devices?* [multiple answers possible] (n=14) | Clinical effectiveness | 100% |
|  | Safety | 93% |
|  | Costs and economic implications | 79% |
|  | Quality of life | 71% |
|  | Organizational aspects (e.g. improved efficiency) | 64% |
|  | Ethical issues | 50% |
|  | Legal issues | 50% |
|  | Wider implications for the patient, relatives, caregivers, and the population (e.g., reducing waiting lists) | 36% |
|  | Social issues | 29% |
|  | Environmental aspects | 29% |
|  | Cultural issues | 14% |
|  | Other, please specify:  - The core aspects are clinical effectiveness, safety and costs. Depending on the intended use and the characteristic of the technology we propose a fit for purpose assessment including other domains (n=1)  - We have a rapid review model (n=1) | 14% |
| *Is the level of evidence different for various types of analyses (e.g., effectiveness, cost-effectiveness, ethics)?* [Open question] (n=13) | *“No.”*  *“Not applicable. we focus on the assessment of clinical effectiveness incl. safety only.”*  *“No (but we do not assess cost-effectiveness).”*  *“There is a recognition of different evidence requirements but in practice analyses often involve effectiveness.”*  *“This can happen due to the different studies being included in the different sections”.*  *“For effectiveness and cost-effectiveness, we develop Systematic Review and for ethics, we usually do Scoping reviews.”*  *“In general, the different dimensions have characteristic sources of evidence. To evaluate efficacy and safety, the quality of the available evidence is usually higher than that available, for example, in the case of evaluating ethical or organizational aspects. In the case of economic aspects, we are guided by Cheers to judge the available evidence.”*  *“Yes evidence level can and are often different. Typically evidence thresholds lowers but we apply standard evidence hierarchy approaches adn outline any associated limitations. For our economic evidence we typically develop de novo costs effectiveness models due to scarcity of studies (and their relevance to context) within the published literature.”*  *“We perform analysis using different approximation for each one of the categories. All is available in our manuals. We have specific manual for effectiveness and safety, economic health evaluations, budget impact analysis, qualitative methods and social participation.”*  *“When we perform a Health Technology Review, we only use quality assessment tools according to research design, not subject-specific quality assessment tools.”*  *“Yes, because, for example, the existing literature in the field of ethics is much scarcer than in the field of effectiveness.”*  *“Yes”.(n=2)* | |
| *Do you think that ethical aspects of high-risk medical devices could be assessed according to scientific standards? Please explain why (not).* (n=13) | *“It is possible to evaluate (and even desirable) the ethical aspect of high-risk devices. In our organization, this dimension is not formally evaluated due to the limited time available to prepare the documents”.*  *“Yes” (n=2)*  *“Particular attention should ethical issues when considering using high-risk medical devices, particularly related to patients' consent”.*  *“I think that the scientific standards of ethical aspects of high-risk medical devices would be very different for each country or society, so it seems difficult to establish scientific standards”.*  *“I think that we need a more consensus on how to do it”.*  *“Not that easy, usually there is no evidence in this aspect”.*  *“I agree, but in practice this is done rarely and is only relevant for few technologies”.*  *No, because ethical aspects used to come from study designs as experts consensus, narrative reviews, etc., and therefore we need to use other methodologies/tools to assess them.*  *“The ethical aspect about high risk medical devices can be evaluated under current scientific standards. Currently, the biggest ethical issues in high risk medical devices is the risk of cross-infection and failure of the device. We have the analytical tools in epidemiology, engineering, microbiology and clinical sciences to evaluate all this issues. The second part that is the perception by stakeholders it can be evaluated using current techniques from psychology, bioethics and social sciences”.*  *“It is difficult to analyze the ethical aspects of medical devices by means of the very same scientific standards used to assess clinical effectiveness. The problem is even more fundamental: ethical analysis is quite often considered as an ancillary step and not as a distinctive aspect of assessing high-risk medical device”.*  *“Unsure”.*  *“Yes and No: There are limits insofar as there are often too few scientific publications on this”.* | |
| *Which methodologies do you use for assessing ethical aspects?* [multiple answers possible] (n=14) | We do not assess ethical aspects | 43% |
|  | EUnetHTA Core Model | 36% |
|  | Specifying norms | 29% |
|  | Interactive Health Technology Assessment | 14% |
|  | Other, please specify:  - We have two publication of ethical issues (both in Spanish); Methodological guideline for ethical issues and a checklist; (n=1)  - Scoping review of the literature (n=1) | 14% |
|  | Coherence analysis (Wide Reflective Equilibrium) | 7% |
|  | Casuistry | 7% |
|  | The Socratic (axiological) approach | 7% |
|  | Constructive technology assessment | 7% |
